# ARTS mediates apoptosis and regeneration of the intestinal stem cell niche

**DOI:** 10.1038/s41467-018-06941-4

**Published:** 2018-11-02

**Authors:** Elle Koren, Yahav Yosefzon, Roi Ankawa, Despina Soteriou, Avi Jacob, Alexander Nevelsky, Rahamim Ben-Yosef, Gil Bar-Sela, Yaron Fuchs

**Affiliations:** 1 Laboratory of Stem Cell Biology and Regenerative Medicine, Department of Biology, Technion Israel Institute of Technology, Haifa, 3200003 Israel; 2 Lorry Lokey Interdisciplinary Center for Life Sciences and Engineering, Technion Israel Institute of Technology, Haifa, 3200003 Israel; 3 Technion Integrated Cancer Center, Technion Israel Institute of Technology, Haifa, 3200003 Israel; 4 Faculty of Life Sciences, Bar-Ilan University, Ramat Gan, 5290002 Israel; 5 Oncology Division, Rambam Health Care Campus, P.O.B. 9602, Haifa, 31096 Israel

## Abstract

Stem cells (SCs) play a pivotal role in fueling homeostasis and regeneration. While much focus has been given to self-renewal and differentiation pathways regulating SC fate, little is known regarding the specific mechanisms utilized for their elimination. Here, we report that the pro-apoptotic protein ARTS (a *Septin4* isoform) is highly expressed in cells comprising the intestinal SC niche and that its deletion protects Lgr5^+^ and Paneth cells from undergoing apoptotic cell death. As a result, the *Sept4/*ARTS^−/−^ crypt displays augmented proliferation and, in culture, generates massive cystic-like organoids due to enhanced Wnt/β-catenin signaling. Importantly, *Sept4/*ARTS^−/−^ mice exhibit resistance against intestinal damage in a manner dependent upon Lgr5^+^ SCs. Finally, we show that ARTS interacts with XIAP in intestinal crypt cells and that deletion of *XIAP* can abrogate *Sept4/*ARTS^−/−^-dependent phenotypes. Our results indicate that intestinal SCs utilize specific apoptotic proteins for their elimination, representing a unique target for regenerative medicine.

## Abstract (web summary)

The mechanisms regulating intestinal stem cell elimination remain unclear. Here, the authors identify that the pro-apoptotic protein ARTS (a *Septin4* isoform) interacts with XIAP in the intestinal stem cell niche to  regulate stem cell survival during intestinal homeostasis and regeneration.

## Introduction

Adult stem cells (SCs) play an essential role in tissue homeostasis, repair and regeneration due to their distinctive capabilities to self-renew and differentiate^1,2^. These unique cells reside in specialized microenvironments termed the SC niche, where the integration of various signaling cues collectively dictates their fate^3–6^. One system that relies heavily on SCs is the intestinal epithelium, which represents the most rapidly replenishing tissue in mammals^7,8^. Intestinal crypt base columnar (CBC) SCs (ISCs), marked by the Lgr5 protein, reside at the base of the intestinal crypts intercalated between terminally differentiated secretory Paneth cells^7^. Close interaction with Paneth cells represents the ISC niche, since Paneth cells supply essential signals required for Lgr5^+^ ISC maintenance^9^. One key pathway that regulates ISC proliferation and differentiation is the Wnt signaling cascade^10–14^. In the intestinal epithelium, the Wnt3 ligand has been shown to be secreted by Paneth cells^15^, promoting both Lgr5^+^ ISC self-renewal, as well as fueling Paneth cell differentiation^16–19^. In the canonical Wnt/β-catenin pathway, Wnt ligands bind to the transmembrane receptor Frizzled, leading to the stabilization and accumulation of cytosolic β-catenin^20^. Translocation of β-catenin to the nucleus results in the transcription of Wnt target genes, which drive cell proliferation and expansion^11,20^.

While growth pathways governing ISC self-renewal and differentiation have been well described, very little is known regarding the specific molecular mechanisms that govern ISC elimination. A fundamental mechanism for the elimination of undesired and potentially dangerous cells is apoptosis, which culminates in the activation of caspases^21–23^. Since improper caspase activation may yield dire consequences, their activation is tightly controlled by both positive and negative regulators^21,24,25^. One crucial layer of negative regulation involves the inhibitor of apoptosis (IAP) family, which can bind directly to and inhibit caspases^26–28^. Perhaps the most potent endogenous caspase inhibitor is the X-linked IAP (XIAP) protein, which functions as an E3 ubiquitin ligase responsible for the degradation of pro-death factors^25,26^. In cells committed to die XIAP must be inactivated by a family of IAP antagonists^22^. One particular mammalian IAP antagonist is ARTS, which is derived from the *Septin4* (*Sept4*) gene by alternative splicing^24,29–32^. Upon induction of the apoptotic cascade, ARTS translocates from the mitochondria to the cytosol where it binds to and antagonizes XIAP^24,30,32–34^. Interestingly, ARTS has been shown to specifically promote apoptosis of hematopoietic and hair follicle SCs in in vivo settings^35,36^.

Given the considerable dependency on SC functionality in the intestinal epithelium, we sought to understand whether ARTS is utilized for the elimination of ISCs and  if it plays a role in ISC-dependent processes. Here we report that ARTS expression is heightened in cells of the intestinal crypt, including Lgr5^+^ ISCs and Paneth cells. We find that whole-body deletion of *Sept4*/ARTS equips the ISC niche with increased resistance against apoptosis and confers protection against intestinal damage in a SC-dependent fashion.

As a consequence of apoptotic resistance, *Sept4*/ARTS^−/−^ mice exhibit augmentation of the ISC niche, as well as enhanced activity of the Wnt/β-catenin pathway. Importantly, we show that loss-of-ARTS-mediated apoptotic resistance is not dependent upon Wnt signaling. Finally, we determine that during apoptosis, ARTS is able to bind to XIAP in the intestinal crypt in vivo and that loss of XIAP abrogates *Sept4*/ARTS^−/−^-dependent phenotypes. These findings reveal an apoptotic module utilized exclusively by the ISC niche, which plays an essential role in governing homeostatic tissue maintenance and regeneration.

## Results

### ARTS regulates expansion of the intestinal SC niche

**Fig. 1:**
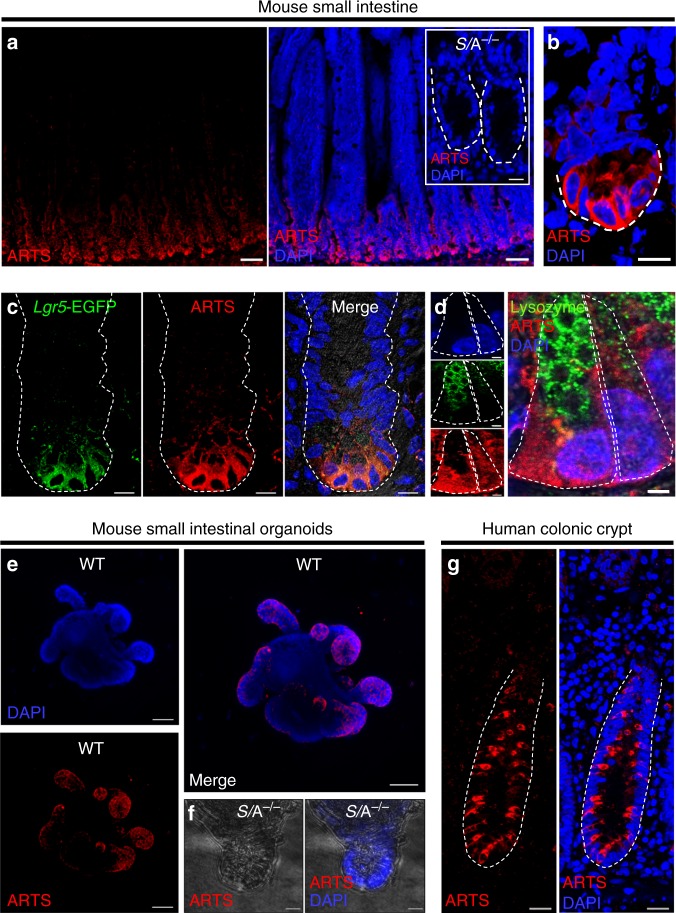
Expression of ARTS is enhanced in intestinal crypt cells. **a** Immunofluorescence (IF) using an antibody specifically against the *Sept4* isoform ARTS reveals high expression in wild-type (WT) intestinal crypts. Inset shows *Sept4*/ARTS^−/−^ (*S*/A^−/−^) control crypts stained for ARTS. **b** Image of single zoomed-in crypt housing ARTS^+^ cells at the crypt base. Dashed white line demarcates crypt base. **c** Single mouse small intestinal crypt stained for *Lgr5*-EGFP^+^ intestinal stem cells (ISCs) and ARTS. **d** Zoom-in of two crypt base cells including lysozyme^+^ Paneth cell and lysozyme^−^ crypt base columnar (CBC) cell stained for ARTS. **e** Mouse small intestinal organoid at high passage stained for ARTS shows heightened expression in de novo organoid crypts. **f** *S*/A^−/−^ negative control organoid crypt stained for ARTS. **g** Normal human colonic crypt shows an alternating pattern of ARTS^+^ cells in the crypt. Images are representative of *n* = 3 human colons, *n* = 3 mice or well triplicates of organoids generated from 3 pooled mouse intestines. White dashed lines demarcate whole intestinal crypts or crypt base cells, unless otherwise indicated. All experiments were repeated at least twice. Scale bars: 5 µm (**d**), 10 µm (**a** inset, **b**, **c**, **f**), 20 µm (**g**), 50 µm (**a**, **e**)

To determine the distribution of ARTS in the mouse intestine, we performed immunofluorescence (IF) utilizing antibodies raised specifically against the ARTS isoform of the *Sept4* gene. We could detect positively stained cells in the small intestinal crypt, with heightened expression in the crypt base (Fig. 1a, b). As expected, we did not detect positively stained cells in control intestines deleted for *Sept4* (Fig. 1a inset). In co-labeling IF experiments we could detect ARTS expression in both Lgr5^+^ ISCs and lysozyme^+^ Paneth cells (Fig. 1c, d). Additionally, we  observed ARTS in wild-type (WT) ex vivo intestinal organoids, which showed a similar expression pattern in crypts (Fig. 1e, f). Extending our analyses to human tissue, we could also distinguish ARTS^+^ cells spanning throughout healthy human colonic crypts (Fig. 1g).

**Fig. 2:**
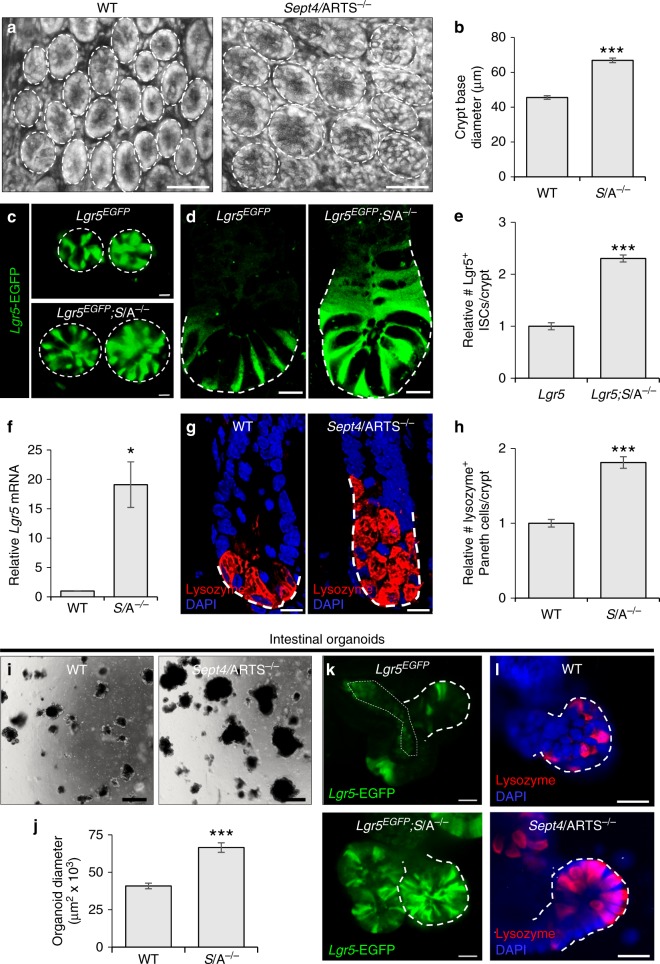
ARTS regulates expansion of the intestinal stem cell niche. **a** Control and *Sept4*/ARTS-deleted (*S*/A^−/−^) small intestinal wholemounts, viewed from the base of the crypt. Note the high presence of granularity in *S*/A^−/−^ crypts, indicative of Paneth cells. Dashed white circles demarcate the crypt base circumference. **b** Quantification for wild-type (WT) and *S*/A^−/−^ crypt base diameter measured from wholemount tissues. **c** Images of the crypt base in reporter mice shows enhanced numbers of *Sept4*/ARTS-deficient *Lgr5*-EGFP^+^ (*Lgr5*^*EGFP*^; *S*/A^−/−^) intestinal stem cells (ISCs). Dashed white circles demarcate the crypt base circumference. **d** Longitudinal sections of small intestinal crypts show that the *Lgr5*^*EGFP*^*; S*/A^−/−^ crypt houses increased numbers of *Lgr5*-EGFP^+^ ISCs. Dashed white line demarcates the crypt base to the highest point of *Lgr5*-EGFP signal. **e** Relative number of *Lgr5*-EGFP^+^ ISCs per intestinal crypt in control and *Lgr5*^*EGFP*^; *S*/A^−/−^ mice. **f** Real-time (RT)-PCR shows increased relative *Lgr5* mRNA in *S*/A^−/−^ intestinal tissue. **g** WT and *S*/A^−/−^ intestinal crypts stained for lysozyme, marking Paneth cells. Dashed white line demarcates the crypt base to the highest point of lysozyme signal. **h** Relative number of Paneth cells per WT and *S*/A^−/−^ crypt. **i** Brightfield images of organoids grown from isolated WT and *S*/A^−/−^ crypts after 8 days in culture. **j** Quantification for maximum organoid size after 20 days in culture. **k**, **l** *S*/A^−/−^ intestinal organoids display greater numbers of **k** *Lgr5*-EGFP^+^ ISCs and **l** lysozyme^+^ Paneth cells in organoid crypts. Dashed white lines demarcate organoid crypts. Images and quantitations are representative of *n* = 3 mice per genotype. All organoid experiments were performed on well triplicates generated from 3 pooled mice per genotype. RT-PCR experiments were performed on 3 pooled mice per genotype, analyzed in triplicates. *P* values were determined using two-tailed unpaired Student’s *t* test where *P < 0.05 and ***P < 0.001. Error bars represent ±s.e.m. All experiments were repeated at least twice. Scale bars: 10 µm (**c**, **d**, **g**), 20 µm (**k**, **l**), 50 µm (**a**), 500 µm (**i**)

Utilizing knockout mice deleted for the *Sept4* locus that encodes for ARTS^36,37^ (denoted *Sept4*/ARTS^−/−^), we set out to examine whether lack of *Sept4*/ARTS could affect intestinal architecture. In contrast to WT mice, *Sept4*/ARTS^−/−^ mice displayed increased crypt diameter, length and cell number (Fig. 2a, b and Supplementary Fig. 1a, b). Of note, we found no significant difference in nuclei size or villi length (Supplementary Fig. 1c–e), indicating that ARTS plays a role predominantly in crypt regulation.

Considered as a pivotal CBC SC population of the intestine, the Lgr5^+^ ISCs are responsible for daily replenishment of the epithelium^38^. To investigate whether ARTS could be utilized for Lgr5^+^ ISC maintenance, we employed *Lgr5-EGFP-IRES-creERT2* (denoted *Lgr5*^*EGFP*^) constitutive reporter mice, crossed to *Sept4/*ARTS^−/−^ mice (*Lgr5*^*EGFP*^; *Sept4*^−/−^). Examination of intestinal tissue wholemounts, which permits visualization of *Lgr5*-EGFP^+^ ISCs in the crypt base, revealed a dramatic increase in *Sept4*/ARTS-null *Lgr5*-EGFP^+^ ISCs (Fig. 2c, d and Supplementary Fig. 1f, g).

This increase could also be seen even in relatively small crypts, in transverse cross sections of *Lgr5*^*EGFP*^; *Sept4*^−/−^ small and large intestinal tissues (Fig. 2d and Supplementary Fig. 1h).

Quantifying the number of Lgr5^+^ ISCs in the small intestinal and colonic *Lgr5*^*EGFP*^ and *Lgr5*^*EGFP*^; *Sept4*^−/−^ crypts revealed a significant increase in *Sept4*/ARTS-null Lgr5^+^ ISCs (Fig. 2e and Supplementary Fig. 1i). Moreover, performing real-time (RT)-PCR analysis revealed markedly higher *Lgr5* mRNA in *Sept4*/ARTS^−/−^ small intestinal tissue, in contrast to WT (Fig. 2f). Considering the expression pattern of ARTS and the increase in both crypt size and Lgr5^+^ ISC numbers when *Sept4*/ARTS was absent, we next asked whether Paneth cell numbers could also be affected. As a first indication, our wholemount analyses revealed a greater display of granularity in the *Sept4*/ARTS^−/−^ crypt (Fig. 2a and Supplementary Fig. 1f, g), a distinct feature of Paneth cells, which are known to harbor distinctly visible and large cytoplasmic granules^39^. Performing IF indicated that again even in relatively smaller crypts, the *Sept4*/ARTS^−/−^ intestinal crypt housed an approximately twofold increase in lysozyme^+^ granular Paneth cells (Fig. 2g, h and Supplementary Fig. 2a, b). Of note, we could also detect increased numbers of Reg4^+^ cells, the Paneth cell equivalent in the colon epithelium (Supplementary Fig. 2c, d).

As a complementary approach, intestinal organoids represent an exceptionally informative ex vivo platform to examine ISC-dependent processes^40,41^. In order to generate intestinal organoids, freshly dissociated crypts were isolated from WT and *Sept4*/ARTS^−/−^ tissues and seeded into a solubilized basement membrane matrix, enabling their growth into three-dimensional intestinal organoids. We found that in agreement with our in vivo findings, *Sept4*/ARTS^−/−^ crypts exhibited greater growth capacity than WT, generating larger organoids up to 20 days in culture (Fig. 2i, j). As expected, intestinal organoids derived from *Lgr5*^*EGFP*^; *Sept4*^−/−^ reporter mice similarly displayed enhanced *Lgr5*-EGFP^+^ ISC numbers, as well as Paneth cell expansion (Fig. 2k, l and Supplementary Fig. 2e, f). Collectively, these data demonstrate that ARTS plays a role in regulating the expansion of Lgr5^+^ ISCs and niche cells both in vivo and ex vivo.

Additionally, we examined whether loss of *Sept4*/ARTS may affect epithelial differentiation. Interestingly, we could detect a significant decrease in Alcian blue^+^ goblet cells in *Sept4*/ARTS^−/−^ tissue (Supplementary Fig. 3a, b), while the numbers of Chromogranin A^+^ enteroendocrine cells appeared unaffected (Supplementary Fig. 3c, d). Taken together our results indicate that *Sept4*/ARTS plays a non-redundant role in regulating cell fate in the intestine.

### *Sept4*/ARTS^−/−^ crypt cells are protected against apoptosis

**Fig. 3:**
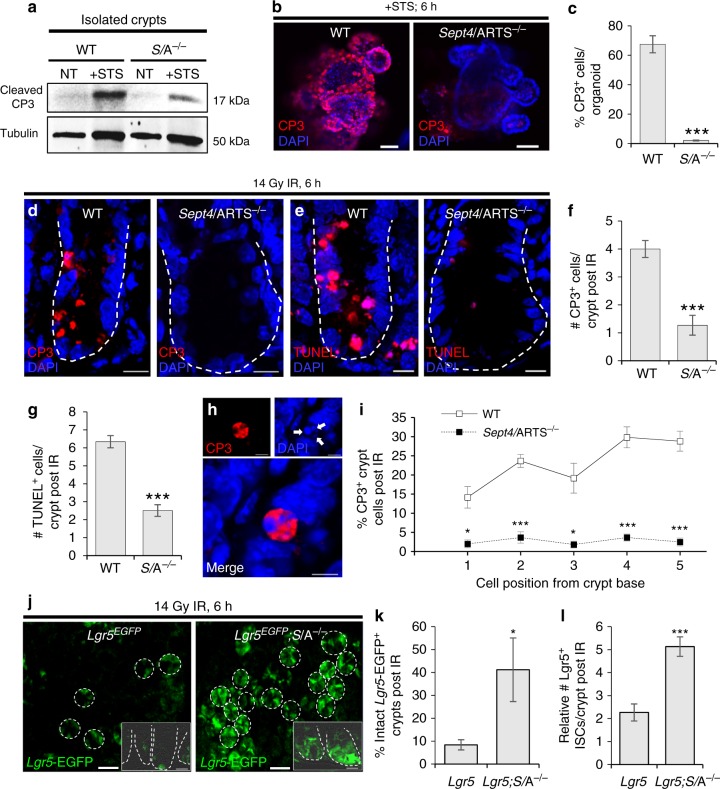
Deletion of *Sept4*/ARTS protects intestinal crypt cells against apoptotic cell death. **a** Western blot of freshly isolated crypts extracted from wild-type (WT) and *Sept4*/ARTS^−/−^ (*S/*A^−/−^) mice [*n* = 3 pooled intestines per genotype], stained for cleaved caspase-3 (CP3) immediately after isolation and after treatment with staurosporine (STS). Tubulin levels were used as a loading control. **b** WT and *S*/A^−/−^ organoids treated with STS and stained for cleaved CP3. **c** Quantification of percentage of cleaved CP3^+^ cells per treated organoid [*n* = 3 wells per treatment of 3 pooled mice per genotype]. **d** Intestinal crypts from WT and *S*/A^−/−^ mice harvested 6 h post 14 Gy abdominal ionizing irradiation (IR), stained for **d** cleaved CP3 and **e** terminal deoxynucleotidyl transferase (TdT) dUTP nick-end labeling (TUNEL). **f**, **g** Quantifications for number of **f** cleaved CP3^+^ and **g** TUNEL^+^ cells displaying apoptotic morphology per crypt at 6 h post IR. **h** Magnified image of a bona fide cleaved CP3^+^ apoptotic crypt cell exhibiting nuclear condensation (white arrows), a classical hallmark of apoptotic cell death. **i** Quantification for percentage of cleaved CP3^+^ crypt cells post irradiation at positions 1–5 from the crypt base, where the first two lowermost crypt base cells were designated position “1” [*P* values were determined comparing between cell positions using two-tailed unpaired Student’s *t* test, where *P < 0.05 and ***P < 0.001]. **j** Surviving *Lgr5*-EGFP^+^ crypts post irradiation. Inset shows transverse image of IR-treated crypts. Dashed white circles and lines (insets) demarcate the crypt base. **k**, **l** Quantification of intact *Lgr5-*EGFP^+^ crypts post irradiation counted from **k** wholemount tissue and **l** transverse sections. All images and quantitations are representative of *n* = 4 mice per genotype, unless otherwise indicated. *P* values were determined using two-tailed unpaired Student’s *t* test, where *P < 0.05 and ***P < 0.001. Error bars represent ±s.e.m. All experiments were repeated at least twice. Scale bars: 5 µm (**h**), 10 µm (**d**, **e**), 20 µm (**j** inset), 50 µm (**b**), 200 µm (**j**)

We next asked whether expansion of the ISC niche could arise as a result of cells evading programmed cell death. More precisely, we hypothesized that ARTS may be utilized to instruct apoptosis within the ISC niche. It should be noted that although the baseline homeostatic level of spontaneous apoptosis in the intestinal crypt base is constant, it is low and difficult to quantify^38,42,43^. Thus, as a first step, we performed cell death assays utilizing live free-floating intestinal crypts. We extracted both WT and *Sept4*/ARTS^−/−^ intestinal crypts from freshly resected tissue to obtain a crypt-enriched preparation. Similarly, extraction was performed on *Lgr5*^*EGFP*^ reporter mice showing that crypts contained viable *Lgr5*-EGFP^+^ cells (Supplementary Fig. 4a). To assess apoptosis, crypts were administered staurosporine (STS), a potent chemical inducer of apoptosis. To examine the levels of crypt cell apoptosis, we extracted total protein and performed immunoblotting using an antibody against cleaved (active) caspase-3, a key executioner of the apoptotic cascade. As expected, we were barely able to detect a signal from freshly isolated crypt lysates (Fig. 3a), confirming that crypt cell apoptosis occurs at a low rate during homeostasis. However, treatment with STS elevated the level of active caspase-3 considerably in the WT, while *Sept4*/ARTS^−/−^ intestinal crypts showed markedly less cleavage of caspase-3 post treatment (Fig. 3a). In agreement, *Sept4*/ARTS^−/−^ organoids that were treated with STS displayed significantly less cleaved caspase-3^+^ cells than treated WT organoids (Fig. 3b, c and Supplementary Fig. 4b). Furthermore, *Sept4*/ARTS^−/−^ organoids appeared capable of withstanding more stressful conditions. One common supplement to promote the viability of organoids after a freeze-thaw cycle is the Rock inhibitor Y-27632^44^. In the absence of Y-27632, a significantly greater number of viable *Sept4*/ARTS^−/−^ organoids could be detected after 8 days post thawing, in contrast to the WT (Supplementary Fig. 4c, d).

Having observed a striking difference in resistance against apoptotic cell death in both crypts and organoids, we next examined whether loss of *Sept4*/ARTS could offer enhanced protection against intestinal damage in an in vivo setting.

Exposure to ionizing radiation (IR) is known to cause elevated levels of cell death in the intestinal crypt^45,46^. As such, we subjected WT and *Sept4*/ARTS^−/−^ mice to 14 Gy of abdominal irradiation, a treatment reported to robustly induce apoptosis of Lgr5^+^ ISCs^38,47^. To analyze peak apoptosis, we harvested mice at 6 h post IR and performed IF against cleaved caspase-3, as well as terminal deoxynucleotidyl transferase (TdT) dUTP nick-end labeling (TUNEL). In both these analyses we detected a significant decrease in cleaved caspase-3^+^ and TUNEL^+^ apoptotic cells within the *Sept4*/ARTS^−/−^ intestinal crypts in contrast to the WT crypt (Fig. 3d–g and Supplementary Fig. 4e, f). Of note, we did not detect a significant difference in the number of CP3^+^ apoptotic cells spanning along the irradiated villi (Supplementary Fig. 4g). In our analyses we considered bona fide apoptotic cells that were positive for cleaved caspase-3 to concomitantly display classic characteristics of apoptotic cell death, including nuclear fragmentation and condensation (Fig. 3h). Importantly, quantifying apoptosis for positions 1–5 from the base of the crypt revealed that WT mice exhibited 15–30 times more apoptotic cells (Fig. 3i). When we administered 14 Gy abdominal IR on control *Lgr5*^*EGFP*^ and *Lgr5*^*EGFP*^; *Sept4*^−/−^ reporter mice we detected a higher number of intact *Lgr5*-EGFP^+^ crypts post irradiation when *Sept4/*ARTS was absent (Fig. 3j, k), as well as increased numbers of *Sept4*/ARTS^−/−^ *Lgr5*-EGFP^+^ ISCs as seen in transverse sections (Fig. 3j insets, l). Collectively, we provide evidence that loss of *Sept4*/ARTS equips intestinal crypt cells with higher resistance against apoptosis and suggests, in part, that this mechanism serves to limit crypt cell expansion.

We also examined anchorage-dependent apoptosis (anoikis) of terminally differentiated cells at the villi tip, considering that our data suggested increased ISC numbers, but no obvious villi lengthening. Indeed, we could detect more caspase-3^+^ villi tips and a higher number of caspase-3^+^ cells per villus unit in *Sept4*/ARTS^−/−^ intestinal tissue (Supplementary Fig. 5a–c).

In light of these data, we asked whether the increased extrusion of terminally differentiated cells in the *Sept4*/ARTS^−/−^ villi could compensate for enhanced migration along the crypt-villus axis. To investigate this scenario, we performed the BrdU label retention assay and assessed migration of BrdU^+^ cells after a 24-h chase. In accordance, increased migration could be observed in the *Sept4*/ARTS^−/−^ small intestine, including the presence of BrdU^+^ epithelial cells at the villi tip, which was absent in the WT (Supplementary Fig. 5d, e). Interestingly, we could also detect heightened numbers of total BrdU^+^ cells, hinting to a potential role of ARTS in regulating cellular proliferation.

### Loss of ARTS enhances proliferation and Wnt/β-catenin activity

**Fig. 4:**
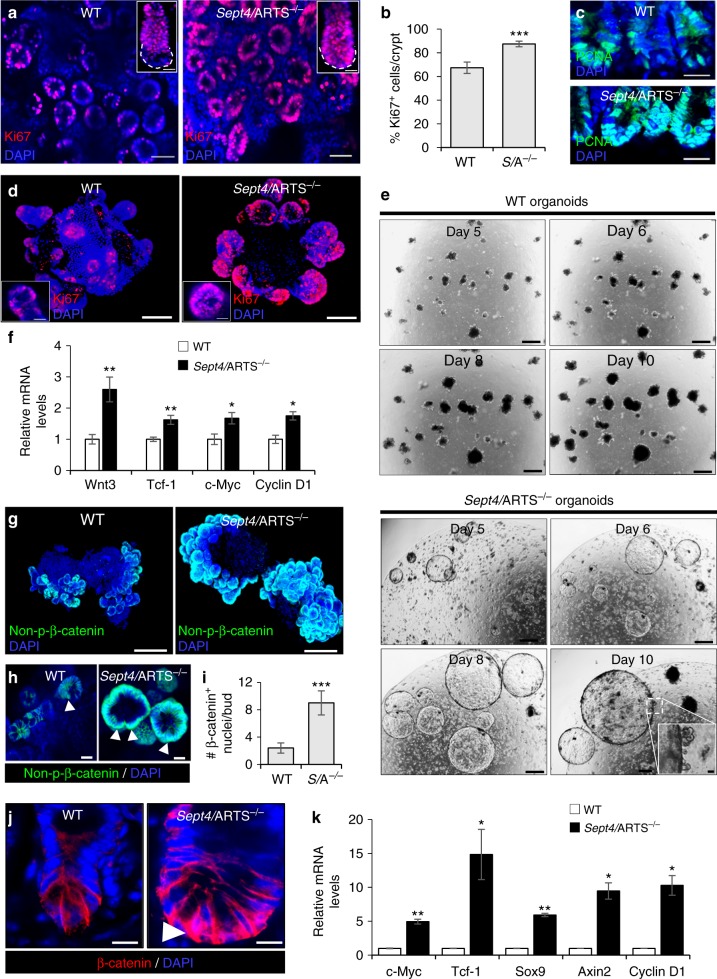
Deletion of *Sept4*/ARTS leads to enhanced activity of the Wnt/β-catenin pathway. **a** Intestinal tissue wholemount stained for Ki67, viewed from the base of the crypt, shows enhanced crypt base proliferation in *Sept4*/ARTS^−/−^ (*S*/A^−/−^) mice. Inset shows isolated and stained crypts, which display greater proliferation in the *S*/A^−/−^ crypt base (dotted white line). **b** Quantification of percentage of Ki67^+^ proliferating cells per crypt. **c** WT and *S*/A^−/−^ intestinal sections stained for PCNA. **d** WT and *Sept4*/ARTS^−/−^ intestinal organoids stained against Ki67. **e** *S*/A^−/−^ organoids often display cystic-like morphology that continue to expand up to 10 days in culture. Inset shows *S*/A^−/−^ cystic organoid with characteristic organoid “buds”. **f ** Real-time (RT)-PCR analysis shows increased relative mRNA transcripts of Wnt3, Tcf-1, c-Myc and Cyclin D1 in control and *S*/A^−/−^ organoids. **g** WT and *S*/A^−/−^ intestinal organoids stained against non-phosphorylated β-catenin. **h** Zoom-in of intestinal organoids shows nuclear β-catenin^+^ cells (white arrowheads) within the *S*/A^−/−^ organoid crypt. **i** Quantifications for number of nuclear β-catenin^+^ cells per organoid crypt. **j** Small intestinal crypts in vivo stained for β-catenin show high nuclear localization (white arrowhead) in the *Sept4*/ARTS^−/−^ crypt base. **k** RT-PCR analysis indicates increased relative mRNA levels of Wnt target genes *c-Myc*, *Tcf-1*, *Sox9*, *Axin2* and *Ccnd1* in *S*/A^−/−^ intestinal tissue [*n* = 3 pooled mice analyzed in triplicates]. Images and quantitations are representative of *n* = 3 mice per genotype. All experiments utilizing organoids were performed on well triplicates generated from 3 pooled mice per genotype. RT-PCR experiments were performed on 3 pooled mice per genotype, analyzed in triplicates. *P* values were determined using two-tailed unpaired Student’s *t* test where *P < 0.05, **P < 0.01 and ***P < 0.001. Error bars represent ±s.e.m. All experiments were repeated at least twice. Scale bars: 10 µm (**h**, **d** insets, **j**), 20 µm (**a** insets, **c**), 50 µm (**a**), 100 µm (**d**, **g**, **e** inset), 500 µm (**e**)

Actively dividing Lgr5^+^ ISCs are responsible for daily replenishment of the tissue^38^. Moreover, Paneth cells directly supply essential growth factors that facilitate Lgr5^+^ ISC expansion^10–12,39^. In light of our results, we asked whether the augmentation of the *Sept4*/ARTS^−/−^ ISC niche could also be due to increased proliferation. As a first step, we performed IF staining against Ki67 and proliferating cell nuclear antigen (PCNA), robust markers of proliferation, which indicated enhanced proliferation in *Sept4*/ARTS^−/−^ crypts in vivo (Fig. 4a–c). Moreover, mice deficient for *Sept4*/ARTS displayed hyperproliferation in the crypt base (Fig. 4a inset, c). Similarly, *Sept4*/ARTS^−/−^ intestinal organoids also displayed increased Ki67^+^ cells in the buds (Fig. 4d). Intriguingly, seeded *Sept4*/ARTS^−/−^ crypts often gave rise to massive cystic-like organoids in culture (Fig. 4e). These *Sept4*/ARTS^−/−^ cystic organoids retained differentiation capacity, as demonstrated by budding off the main cystic body and the presence of relatively morphologically normal organoids within the same well (Fig. 4e inset). Furthermore, after approximately 10 days in culture, many cystic *Sept4*/ARTS-deficient organoids became largely differentiated, collapsing into organoids that displayed regular morphology (Supplementary Fig. 6a). Of note, we could detect Paneth cells, as well as neighboring CBC cells in cystic organoid buds (Supplementary Fig. 6b, c).

One distinct signaling pathway responsible for coordinating proliferation within the intestinal crypt is the Wnt/β-catenin pathway. Cystic organoids are often seen as a result of administering exogenous recombinant Wnt3a^9^, or can develop from ISCs harboring mutant *APC*, a key negative regulator of the Wnt pathway^48,49^. As such, this particularly striking result is highly indicative that deletion of *Sept4*/ARTS manifests in Wnt pathway overactivation.

In ex vivo intestinal organoids Paneth cell-derived Wnt3 has been shown to function as a critical signal for ISC maintenance^39^. Performing RT-PCR analysis indicated a significant ~2.5-fold increase in Wnt3 transcript levels in *Sept4*/ARTS^−/−^ organoids (Fig. 4f). In complement, we could also detect higher mRNA levels of the Wnt target genes *Tcf-1*, *c-Myc* and *Ccnd1* (Fig. 4f). During the cascade, as a result of Wnt ligand binding, β-catenin is stabilized and translocates to the nucleus. Performing IF against non-phosphorylated (active) β-cateninrevealed  higher levels in *Sept4*/ARTS^−/−^ intestinal organoids (Fig. 4g). Importantly, a greater number of *Sept4*/ARTS^−/−^ organoid crypt cells displayed nuclear (active) non-phosphorylated β-catenin than WT organoids (Fig. 4h, i).

We also examined β-catenin in vivo, which revealed heightened nuclear localization in the *Sept4*/ARTS^−/−^ crypt base, indicative of higher Wnt pathway activity (Fig. 4j). In agreement, RT-PCR analysis of Wnt pathway target genes *c-Myc*, *Tcf-1*, *Ccnd1*, *Axin2* and *Sox9* revealed between 3- and 14-fold increases in relative mRNA levels in the *Sept4*/ARTS^−/−^ intestinal tissue (Fig. 4k). Of note, the *Lgr5* SC marker, which is also a classic Wnt pathway target gene in the intestine, was significantly increased in *Sept4*/ARTS^−/−^ intestinal tissue (Fig. 2f).

Additionally, we isolated control and *Sept4*/ARTS^−/−^ *Lgr5*-EGFP^+^ ISCs via fluorescence-activated cell sorting (FACS), which could give rise to *Lgr5*-EGFP^+^ reporter organoids in culture (Supplementary Fig. 7a, b). In accordance with our previous results, we detected an approximately twofold increase in the numbers of isolated *Sept4*/ARTS^−/−^ *Lgr5*-EGFP^+^ ISCs (Supplementary Fig. 7a). We next performed RT-PCR and could detect higher mRNA levels of the Wnt target genes *c-Myc* and *Ccnd1* in isolated *Sept4*/ARTS^−/−^ ISCs, indicating that Wnt target genes were not simply increased in the tissue due to greater numbers of Wnt-addicted cells (Supplementary Fig. 7c).

Collectively, these data indicate that expansion of the ISC niche, mediated by loss of ARTS, manifests in enhanced proliferation and Wnt/β-catenin activity.

### ARTS mediates apoptosis independently of Wnt signaling

The intestinal crypt in vivo receives Wnt signals from both epithelial Paneth cells and the underlying mesenchyme^16^. Thus, one possibility is that a non-epithelial, niche-like cell population could contribute to the enhanced Wnt signaling in the *Sept4*/ARTS^−/−^ intestinal tissue. Should the observed increase in Wnt activity and expansion in *Sept4*/ARTS^−/−^ intestine be supported by such a mesenchymal population, we could anticipate that epithelial phenotypes would be of a transient nature in an ex vivo organoid setting.

**Fig. 5:**
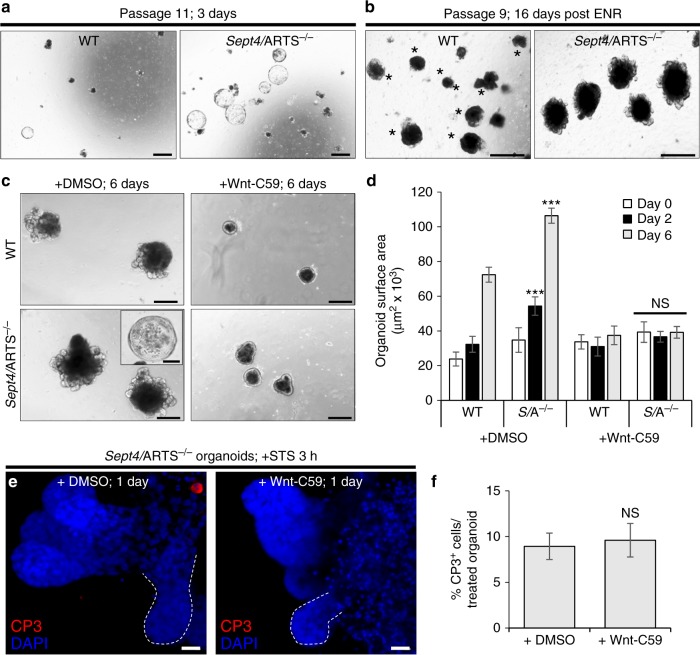
*Sept4*/ARTS^−/−^-mediated apoptotic resistance in organoids is not dependent upon Wnt secretion. **a** *Sept4*/ARTS^−/−^ (*S*/A^−/−^) organoids continue generating cysts at high passage. **b** After 16 days post splitting wild-type (WT) and *S*/A^−/−^ organoids at passage 8, and without new supplementation of basic ENR (EGF, Noggin, R-Spondin1) organoid media, *S*/A^−/−^ organoids retain larger size and remain viable in contrast to WT organoids. Asterisks indicate dying organoids lacking viable crypts. **c** WT and *S*/A^−/−^ intestinal organoids treated with DMSO (control) or Wnt-C59 Porcupine inhibitor for up to 6 days. Both WT and *S*/A^−/−^ organoids inhibited for Wnt displayed similar characteristics including differentiation, shrinkage  and loss of *S*/A^−/−^ cysts. **d** Quantification for organoid size during treatment indicates that both WT and *Sept4*/ARTS^−/−^ organoids are equally dependent on paracrine Wnt signaling for their function [*P* values were determined between DMSO or C59-treated WT and *Sept4*/ARTS^−/−^ using two-tailed unpaired Student’s *t* test, where ***P < 0.0005 and *P* > 0.05 indicates no significance (NS)]. **e** *S*/A^−/−^ organoids treated with staurosporine (STS; control) and co-treated with STS and Wnt-C59 inhibitor, stained against cleaved caspase-3 (CP3). Dashed white line demarcates organoid crypt. **f** Percentage of cleaved CP3^+^ cells per treated organoid [NS was determined using two-tailed unpaired Student’s *t* test where *P* > 0.05]. Images and quantitations are representative of organoids generated from *n* = 3 pooled mice per genotype, analyzed in well triplicates. Error bars represent ± s.e.m. All experiments were repeated at least twice. Scale bars: 10 µm (**e**), 200 µm (**c, c** inset), 500 µm (**a**, **b**)

As such, we continuously passaged intestinal organoids and found that after several passages *Sept4*/ARTS^−/−^ organoids still retained the expanded niche, displayed cystic morphology and exhibited dramatic expansion capacity at high passage number (Fig. 5a and Supplementary Fig. 8a–f). Moreover, at high passage, the *Sept4*/ARTS^−/−^ organoids retained larger size and showed significantly more viable buds than WT after 16 days when fresh (epidermal growth factor (EGF), Noggin, R-Spondin1; ENR) media was not replaced (Fig. 5b). As such, our data indicate that the observed *Sept4*/ARTS^−/− ^phenotypes are propagated via epithelial signaling.

In contrast to the small intestine, the colon crypt does not house Paneth cells. As such, a requirement for the establishment of colon organoids is the supplementation of recombinant Wnt3a^44^ (Supplementary Fig. 9a). Surprisingly, we found that seeded *Sept4*/ARTS^−/−^ colonic crypts could give rise to colonoids in the absence of Wnt3a (Supplementary Fig. 9b), possibly due to increased intrinsic Wnt activation. Therefore, we set out to investigate whether the expansion phenotypes conferred by the deletion of *Sept4*/ARTS is dependent upon Wnt secretion. For this aim we utilized the Wnt-C59 Porcupine inhibitor, which prevents the secretion and activity of Wnt ligands^50^. We examined whether administration of Wnt-C59 could attenuate the formation of colonoids. Our data clearly show that colonoid establishment of both WT and *Sept4*/ARTS^−/−^ was severely abrogated, indicating that ARTS does not function as an intrinsic inhibitor of the Wnt signaling cascade in this setting (Supplementary Fig. 9c). Similar results could be attained in Wnt-inhibited small intestinal WT and *Sept4*/ARTS^−/−^ organoids, which displayed highly comparable morphology including loss of crypts and shrinkage after a prolonged period (Fig. 5c, d).

These data could suggest that the apoptotic resistance conferred by loss of ARTS depends upon increased Wnt activity. In order to examine this possibility, we administered the Wnt-C59 inhibitor to organoids for 24 h. We determined that this reduced timeframe sufficiently attenuated Wnt pathway activity as indicated by decreased Wnt target gene transcript levels (Supplementary Fig. 9d). Additionally, a less dramatic effect could be seen on organoid morphology, as demonstrated by the retention of crypts (Fig. 5e). After 24 h, we treated control and Wnt-inhibited organoids with STS and examined the activation of caspase-3. Our results indicate that inhibition of Wnt activity does not affect the apoptotic resistance of *Sept4*/ARTS^−/−^ organoids (Fig. 5e, f).

In conclusion, our results suggest that the increase in Wnt activity displayed by *Sept4*/ARTS^−/−^ organoids plays a critical role in organoid development, but does not significantly influence the apoptotic resistance conferred by loss of ARTS.

### *Sept4*/ARTS^−/−^ mice are protected against intestinal wounding

**Fig. 6:**
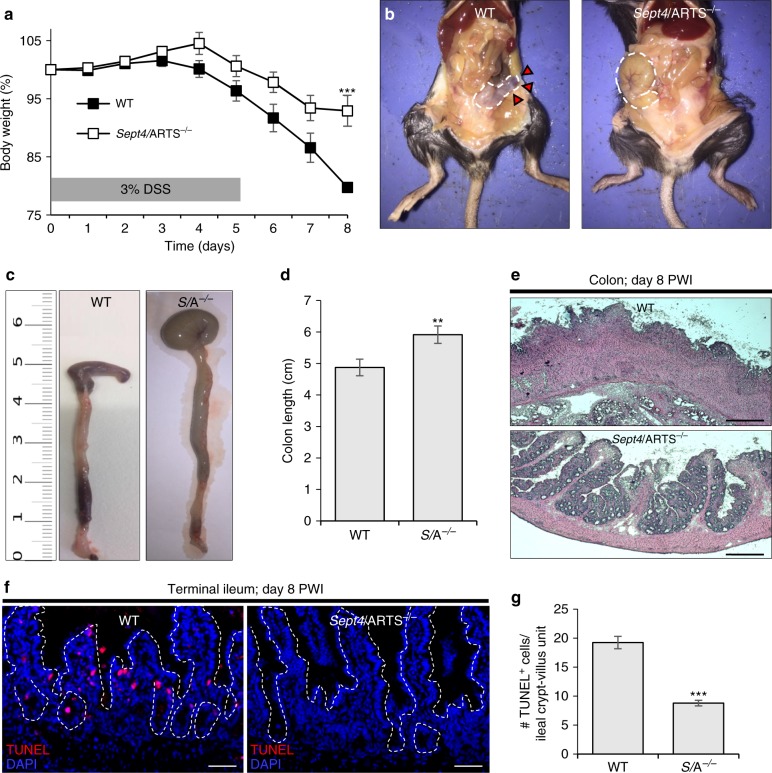
Mice lacking *Sept4*/ARTS exhibit enhanced resistance against intestinal wounding. **a** Body weight (%) of wild-type (WT) and *Sept4*/ARTS^−/−^ (*S*/A^−/−^) mice depicted over days 0–8 of dextran sodium sulfate (DSS; 3% w/v) administration [***P value < 0.002 was determined by comparing the same time point using unpaired two-tailed Student’s *t* test]. **b** WT and *S*/A^−/−^ mice sacrificed at day 8 post wound infliction (PWI). Dissection through the abdominal wall reveals that the *S*/A^−/−^ cecum of the large intestine retains regular shape, size and color, in contrast to the WT (red arrows). Dashed white line indicates cecum. **c** Extracted colons from WT and *S*/A^−/−^ mice at day 8 PWI. Length is in cm. **d** Quantification of colon length (cm) PWI. **e** Hematoxylin and eosin (H&E) staining of resected colon tissue at day 8 PWI. **f** WT and *S*/A^−/−^ terminal ileum at day 8 PWI stained for terminal deoxynucleotidyl transferase (TdT) dUTP nick-end labeling (TUNEL)^+^ apoptotic cells. Dashed white lines demarcate crypt-villi units. **g** Quantifications of number of TUNEL^+^ apoptotic cells per crypt-villus unit post DSS treatment [*n* = 3 mice per genotype]. **P < 0.01 and ***P < 0.005 were determined by unpaired two-tailed Student’s *t* test, unless otherwise specified. Images and quantitations are representative of *n* = 11 mice per genotype unless otherwise indicated. Error bars represent ±s.e.m. All experiments were repeated at least twice. Scale bars: 50 µm (**f**), 200 µm (**e**)

Given the striking expansion of *Sept4*/ARTS-deficient Lgr5^+^ ISCs and Paneth cells, and considering their fundamental roles in regeneration and barrier maintenance, we next asked whether loss of *Sept4*/ARTS could protect mice against intestinal barrier damage or increase their capacity for wound repair. To this end, we administered 3% (w/v) dextran sodium sulfate (DSS) to WT and *Sept4*/ARTS^−/−^ mice, a treatment that robustly inflicts damage to the mouse intestine, resulting in acute inflammation and symptoms of ulcerative colitis^51^. We found that *Sept4*/ARTS^−/−^ mice displayed markedly decreased weight loss over the course of the treatment (Fig. 6a). Remarkably, at termination of the experiment we noted that *Sept4*/ARTS^−/−^ mice also exhibited distinctly less macroscopic damage to the cecum (Fig. 6b) and displayed little to no colorectal bleeding, which represents a classic symptom of the DSS-injury model (Fig. 6b, c). Colon shortening, another characteristic of DSS-induced colitis, was also less pronounced in *Sept4*/ARTS^−/−^ mice (Fig. 6b–d). Furthermore, histological examination of the damaged colon tissue demonstrated greater crypt loss in the WT, as well as thickening of the lamina propria, while normal colonic crypt architecture was largely maintained in *Sept4*/ARTS^−/−^ mice (Fig. 6e).

While damage caused by DSS is largely reported in the colon, we also observed macroscopic damage to WT terminal ileum, as demonstrated by pallid tissue color and the presence of soft and bloody stool. When we performed TUNEL staining on terminal ileum, we could detect a significant amount of TUNEL^+^ cells in the WT (Fig. 6f, g). Moreover, TUNEL analysis indicated higher resistance against cell death in the *Sept4*/ARTS^−/−^ mice following DSS treatment (Fig. 6f, g). Of note, more apoptotic cells could be seen in the DSS-treated villi of WT mice, whereas in *Sept4*/ARTS^−/−^ these were largely lacking. These results suggest that enhanced survival of crypt cells, as a result of *Sept4*/ARTS deletion, confers protection against detrimental intestinal barrier loss, preventing widespread cell death and colitis-like disease symptoms.

### Contribution of *Sept4*/ARTS^−/−^ SCs to epithelial repair

**Fig. 7:**
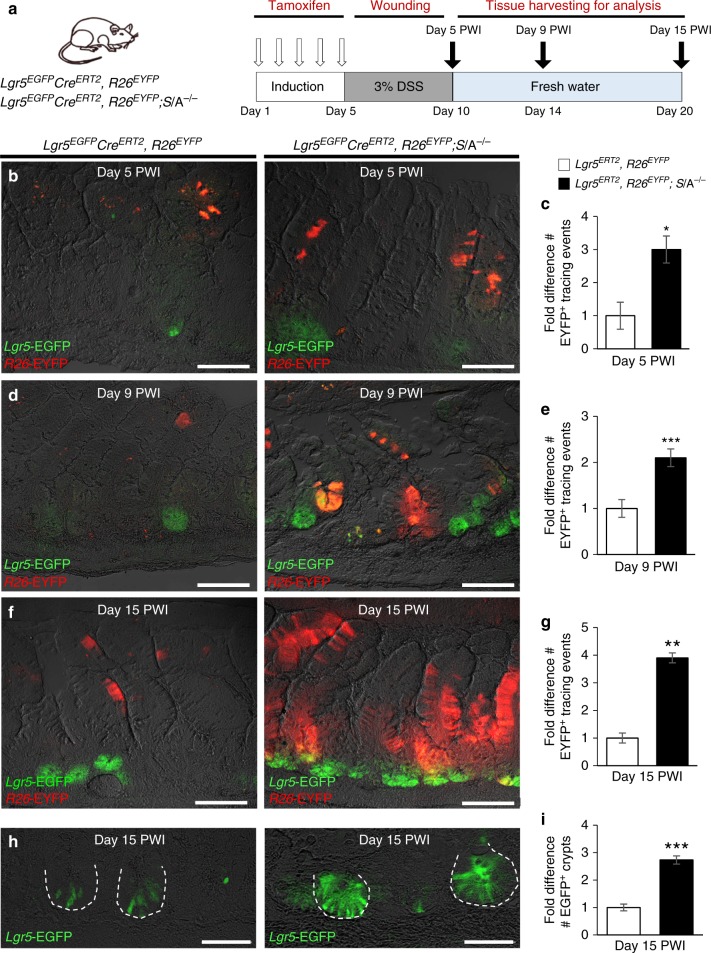
*Sept4*/ARTS^−/−^ mice display increased intestinal regeneration post wounding. **a** Experimental scheme for transgenic lineage tracing mice, tamoxifen administration, dextran sodium sulfate (DSS)-mediated intestinal wounding and tissue harvesting. **b**–**g** Images and quantifications for lineage tracing in control *Lgr5*^*EGFP*^*Cre*^*ERT2*^, *R26*^*EYFP*^ (denoted *Lgr5*^*ERT2*^, *R26*^*EYFP*^) and *Lgr5*^*EGFP*^*Cre*^*ERT2*^, *R26*^*EYFP*^; *S*/A^−/−^ (*Lgr5*^*ERT2*^, *R26*^*EYFP*^; *S*/A^−/−^) terminal ileal tissues at **b**, **c** 5 days, **d**, **e** 9 days and **f**, **g** 15 days post wound infliction (PWI). Images show *Lgr5*-EGFP^+^ intestinal stem cells (ISCs) and their progeny expressing enhanced yellow protein (EYFP) under the *Rosa26* (*R26*) promoter (*R26-*EYFP^+^) during the post-wounding regenerative response. Quantifications show fold difference in number of tracing events per time point PWI. **h**, **i** Images of **h** *Lgr5*-EGFP^+^ crypts in terminal ileum at 15 days PWI and **i** quantifications of fold difference in number of regenerated EGFP^+^ crypts at 15 days PWI. Dashed white line demarcates crypts. All images and quantifications are representative of *n* = 3 mice per genotype per group. *P* values were determined by unpaired Student’s *t* test, where *P < 0.05, **P < 0.01 and ***P < 0.005. Error bars represent ± s.e.m. All experiments were repeated twice. Scale bars: 50 µm (**h**), 100 µm (**b**, **d**, **f**)

Given that *Sept4*/ARTS^−/−^ mice displayed a significant increase in ISC expansion as well as resistance to DSS wounding, we next examined the contribution of Lgr5^+^ ISCs to the repair process. To this end, we utilized *Lgr5-EGFP-IRES-creERT2-R26-EYFP* (*Lgr5*^*EGFP*^*Cre*^*ERT2*^, *R26*^*EYFP*^) and crossed *Lgr5-EGFP-IRES-creERT2-R26-EYFP*; *Sept4*/ARTS^−/−^ (*Lgr5*^*EGFP*^*Cre*^*ERT2*^, *R26*^*EYFP*^; *Sept4*^−/−^) lineage tracing mice. Following the administration of tamoxifen, which permits conditional and inducible single color (enhanced yellow protein; EYFP) lineage tracing of Lgr5^+^ ISCs and their progeny, we employed a 5-day 3% (w/v) DSS/15-day recovery regime (Fig. 7a). Strikingly, mice deleted for *Sept4*/ARTS displayed greater numbers of EYFP^+^ clonal tracing events than the control throughout the regenerative response (Fig. 7b–g). Specifically, on day 9 post wound infliction (PWI), large trails of *Sept4*/ARTS^−/−^ EYFP^+^ clones could be seen emanating from *Lgr5*-EGFP^+^ crypts, which were largely absent in the control (Fig. 7d, e). On day 15 PWI, larger and more numerous trails of *Sept4*/ARTS^−/−^ EYFP^+^ clones could be observed in the terminal ileum in comparison to the control (Fig. 7f, g), as well as larger regenerated crypts harboring more *Lgr5*-EGFP^+^ ISCs (Fig. 7h, i). Moreover, examination of the repairing colon revealed a similar pattern, where *Sept4*/ARTS^−/−^ EYFP^+^ clonal ribbons were more abundant at all time points PWI (Supplementary Fig. 10a–d). These findings indicate that lack of ARTS-dependent crypt apoptosis significantly improves Lgr5^+^ ISC-mediated regeneration post wounding and contributes to the enhanced protection seen against DSS wounding.

### XIAP serves as a target of ARTS in the intestinal crypt

In mammalian cells, ARTS has been established as a pro-apoptotic protein that, upon induction of the apoptotic cascade, binds to XIAP and facilitates its degradation, thus leading to activation of the caspases^24,52^. Importantly, in both hematopoietic and hair follicle SCs, loss of *Sept4*/ARTS has been shown to lead to elevated levels of XIAP^35,36^. These reports, and in light of the results presented so far, strongly suggest that ARTS maintains its function as an antagonist of XIAP in the intestinal crypt.

**Fig. 8:**
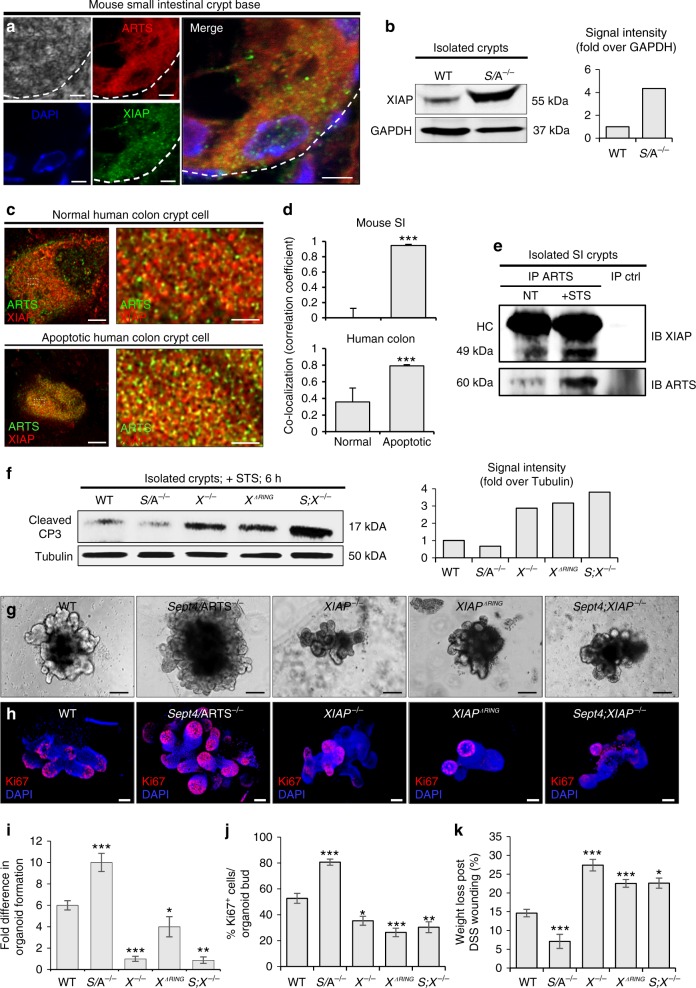
ARTS mediates its functions via interaction with XIAP in the intestinal crypt. **a** Confocal microscopy image of mouse small intestinal crypt base shows co-localization of ARTS and XIAP within the same cell [*n* = 4 mice]. **b** Western blot of XIAP in isolated wild-type (WT) and *Sept4*/ARTS^−/−^ (*S*/A^−/−^) crypts. Signal intensity is normalized to GADPH. **c** Super-resolution stimulated emission depletion (STED) microscopy shows co-localization of ARTS and XIAP in normal and apoptotic human colonic crypt base cells. **d** Pearson’s coefficient values demonstrate higher co-localization of ARTS and XIAP in both mouse and human apoptotic crypt cells [*n* = 3 human colons and *n* = 4 mice. Error bars represent s.e.m.]. **e** Co-immunoprecipitation (co-IP) of ARTS and XIAP in isolated *XIAP*^*ΔRING*^ small intestinal crypts. *XIAP*^*ΔRING*^ only interacts mildly with ARTS in the absence of apoptotic stimulation. After staurosporine (STS) treatment, efficient binding between ARTS and *XIAP*^*ΔRING*^ is detected. **f** Western blot and signal intensity of active caspase-3 (CP3) in STS-treated crypts show that deletion of *XIAP* or *RING* domain increases cleaved CP3 levels. **g** Organoids derived from *XIAP*^−/−^, *XIAP*^*ΔRING*^ and *S;X*^−/−^ crypts display hindered growth and development after 11 days post seeding. **h** *XIAP*^−/−^, *XIAP*^*ΔRING*^ and *S;X*^−/−^ organoids stained for Ki67. **i**, **j** Fold difference in **i** organoid formation capacity and **j** number of Ki67^+^ cells in WT, *S/*A^−/−^, *XIAP*^−/−^, *XIAP*^*ΔRING*^ and *S;X*^−/−^ organoids. **k** *XIAP*^−/−^, *XIAP*^*ΔRING*^ and *S;X*^−/−^ mice display greater body weight loss than both WT and *S*/A^−/−^ mice at 10 days following a 5-day DSS (2.5% w/v) regime [*n* = 3 mice per genotype]. All blots are representative of *n* = 3 pooled mice per genotype. Organoid data are representative of *n* *=* 3 pooled mice per genotype analyzed in well triplicates. *P* values were determined between each group or for each genotype compared to the WT control using unpaired two-tailed Student’s *t* test, where *P < 0.05, **P < 0.01, and ***P < 0.005. Error bars represent ±s.e.m., unless otherwise stated. All experiments were repeated at least twice. Scale bars: 1 µm (**c** right), 3 µm (**c** left), 5 µm (**a**), 100 µm (**g**, **h**)

As a first step, we performed IF for XIAP expression. We could detect relatively high expression of XIAP in the mouse small intestinal crypt (Supplementary Fig. 11a). Similarly, we found that XIAP is expressed in human colon crypts (Supplementary Fig. 11b). We next used confocal microscopy to examine the expression of both ARTS and XIAP and could detect co-expression within the same crypt base cells (Fig. 8a). Such co-labeling was also detected in healthy human crypt cells (Supplementary Fig. 11b).

Notably, when we performed immunoblotting against XIAP from proteins extracted from isolated crypts we could detect heightened XIAP levels when ARTS was absent (Fig. 8b).

Given that our confocal analyses indicated that ARTS and XIAP are both expressed within crypt cells, we next utilized super-resolution stimulated emission depletion (STED) microscopy to examine ARTS and XIAP co-localization. Since ARTS translocates from mitochondria during the apoptotic process and XIAP is located in the cytoplasm, we detected low levels of co-localization between ARTS and XIAP in normal mouse and human colon crypt cells (Fig. 8c, d). In contrast, in both human and mouse crypt cells exhibiting apoptotic morphology, we could identify higher levels of co-localization occurring between these two proteins (Fig. 8c, d and Supplementary Fig. 11c).

Since we could detect co-localization of ARTS and XIAP, we next asked whether they indeed bind within intestinal crypt cells. However, since ARTS and XIAP mutually degrade one another via the ubiquitin proteasome system^32,53^, for these analyses we utilized mice harboring a mutated E3 ligase *RING* domain (*XIAP*^*ΔRING*^), which lacks ubiquitination capacity^33^. Proteins extracted from both freshly isolated and STS-treated *XIAP*^*ΔRING*^ crypts were subjected to co-immunoprecipitation (co-IP). Intriguingly, we could precipitate ARTS dimers, which have been reported to demonstrate higher binding efficiency to XIAP^34^. In untreated crypts we could detect low ARTS levels, while STS treatment induced markedly stronger ARTS levels. This is in agreement with previous findings in other cell types^32^. Here ARTS is clearly seen to efficiently bind the *XIAP*^*ΔRING*^ protein, indicating that the two interact in crypt cells during the apoptotic process (Fig. 8e).

These data suggest that XIAP serves as a target of ARTS and that deletion of *Sept4/*ARTS leads to decreased degradation of XIAP, which sufficiently averts execution of the apoptotic cell death program.

As such, we next examined whether deletion and loss of *XIAP* function could render intestinal crypts more susceptible to apoptosis. To this end, we extracted proteins from isolated WT, *Sept4*/ARTS^−/−^, *XIAP*^−/−^, *XIAP*^*ΔRING*^ and from mice deleted for both *Sept4*/ARTS^−/−^; *XIAP*^−/−^ (*S;X*^−/−^) crypts that were treated with STS. As expected, we could detect markedly higher levels of cleaved caspase-3 in *XIAP*^−/−^, *XIAP*^*ΔRING*^ and *S;X*^−/−^ crypts, in contrast to WT and *Sept4*/ARTS^−/−^ crypts (Fig. 8f). Given that caspase-3 represents a robust downstream target of XIAP, and is regarded a classic marker of apoptosis, these findings demonstrate that ARTS-mediated crypt apoptosis is directed via the anti-apoptotic activity of XIAP.

Extending our analyses to an in vivo setting, we asked whether loss of *XIAP* could reverse the phenotypes observed in *Sept4*/ARTS^−/−^ intestinal tissue. Indeed, we could detect lower numbers of Paneth cells in *XIAP*^−/−^ crypts, as well as diminished proliferation and increased goblet cells in the villi (Supplementary Fig. 12a–f). Moreover, increased numbers of apoptotic caspase-3^+^ cells could be seen in the *XIAP*^−/−^ crypts during baseline homeostasis (Supplementary Fig. 12g, h). Additionally, we observed a striking reversal in growth and proliferation of *XIAP*^−/−^, *XIAP*^*ΔRING*^ and *S;X*^−/−^ seeded crypts, in contrast to the WT and *Sept4*/ARTS^−/−^ organoids after 11 days in culture (Fig. 8g–j).

We next hypothesized that in contrast to *Sept4*/ARTS^−/−^ mice, *XIAP*^−/−^, *XIAP*^*ΔRING*^ and *S;X*^−/−^ mice would display increased susceptibility to DSS treatment. Employing a 5-day 2.5% (w/v) DSS wounding regime, we could detect significant weight loss of *XIAP*^−/−^, *XIAP*^*ΔRING*^ and *S;X*^−/−^ mice, which peaked at 10 days PWI (Fig. 8k). Examination of the terminal ileum PWI revealed exacerbated inflammation, distorted tissue architecture, muscle thickening and greater crypt loss in the *XIAP*^−/−^, *XIAP*^*ΔRING*^ and *S;X*^−/−^ mice (Supplementary Fig. 13a, b).

Taken together, our findings show that ARTS mediates its pro-apoptotic function in the intestinal crypt via its interaction with XIAP, which has critical implications for intestinal homeostasis and regeneration.

## Discussion

Very few studies have examined the manner in which SCs are eliminated, which is critical for controlling SC numbers and preventing the propagation of abnormal progeny. One key pathway responsible for the elimination of cells is apoptosis. In the intestinal epithelium, it has been elegantly shown that apoptosis can differentially affect SC-dependent replenishment and clonogenicity, suggesting that alternative apoptotic mechanisms exist in ISCs to either limit or drive their death^47^.

Our work sheds new light on this topic, describing how the apoptotic ARTS/XIAP module plays a critical role in coordinating cell death specifically within the intestinal crypt, which has critical implications for ISC niche-dependent homeostasis and regeneration. Our findings suggest a predominant role of ARTS within the intestinal crypt and indicates that differentiated epithelial cells do not require ARTS for their elimination. In agreement, it has previously been reported that ARTS regulates apoptosis of hair follicle SCs and hematopoietic stem/progenitor cells^54^. Relating to this, one particularly striking finding from this study is that loss of ARTS was able to confer protection against the induction of experimental colitis in mice. As such, given that barrier loss and immune cell infiltration impact the outcome and severity of DSS wounding, and we utilize a whole-body *Sept4*/ARTS knockout, it is possible that protection from damage was also influenced by immune cells lacking ARTS. Very few studies have examined the changes in and contributions of bone marrow cells. However, there are indications that DSS-treated mice display loss of pre-B and -T cells, including heightened levels of apoptosis in CD4^+^CD8^+^ thymocytes^55^. This may be an interesting observation to consider since it has been reported that *Sept4*/ARTS^−/−^ CD4^+^CD8^+^ cells display greater apoptotic resistance than their WT counterparts^54^.

In this present study we find that as a result of loss of ARTS-mediated apoptosis, the intestinal crypt displays expanded size, as well as augmented Lgr5^+^ ISC and Paneth cell numbers. Considering the vital role of Paneth cells as niche cells, we expected that their expansion would result in elevated levels of growth pathway signaling. One interesting observation from this study is that less goblet cells could be seen in the *Sept4/*ARTS^−/−^ epithelium. Interestingly, high Wnt signaling has been described to interfere with goblet cell differentiation, while its inhibition directs differentiation toward the goblet cell fate^56,57^. In this regard, it has been shown that a high degree of negative crosstalk exists between the Wnt and Notch signaling pathways, to control the balance between ISC renewal and differentiation^58^. Given the vast contributions of these key cascades toward many processes, including development and disease, future work should examine the extent that ARTS-mediated apoptosis can affect Wnt, and possibly also Notch signaling, in various biological settings.

Initially this study suggested a “chicken-or-egg”-like causality dilemma, where one might ask what comes first: resistance against apoptosis or Wnt pathway overstimulation? Both modules represent fundamental contributors to cell pool expansion. However, we found that transient inhibition of the Wnt pathway did not abrogate the apoptotic resistance observed in *Sept4*/ARTS^−/−^ organoids. Thus, the most likely scenario is that upon loss of ARTS, diminished apoptosis causes ISC and niche cell longevity, thus augmenting Wnt activation, which concomitantly creates an amplification loop promoting ISC expansion and Paneth cell differentiation. Nevertheless, it is still possible that since the Wnt/β-catenin pathway also regulates the transcription of various genes involved in the control of apoptosis^59^, its overactivity could further heighten the apoptotic resistance of *Sept4*/ARTS^−/−^ crypt cells.

The manipulation of apoptosis in SCs can have critical implications for SC-dependent processes including epithelial restitution. In this study, we found that deletion of *Sept4*/ARTS was able to confer protection against both radiation-induced cell death and DSS-mediated wounding. These results could have direct clinical implications for limiting barrier loss in intestinal bowel diseases. Additionally, aggressive chemo-radiotherapies often result in lethal tissue injury, particularly to the intestine. In this study we show that ARTS is also expressed in human colonic crypts, thus offering significant translational potential. Our results suggest that cancer treatments, coupled with the transient inhibition of ARTS activity, may serve as a powerful approach for limiting ISC death and preventing toxicity effects in patients.

However, while less apoptosis resulted in elevated SC numbers, which is desirable for restoring damaged tissue, expanded SC numbers may heighten the possibility for tumorigenesis. We surmise that loss of ARTS function, over an extended period of time, may promote malignant transformation as well as confer protection to transformed SCs, thus potentiating their survival and pro-tumorigenic activity^29^. In support of this, it has been previously reported that *Sept4*/ARTS^−/−^ mice exhibit enhanced tumor susceptibility^54^. Over recent years, Lgr5^+^ cancer SCs (CSCs) have emerged as fundamental drivers of intestinal tumorigenesis^60,61^. In intestinal adenomas, Lgr5^+^ CSCs have been found to maintain contact with Paneth cells to fuel adenoma development and replenishment^60^. In this regard, aberrant overactive Wnt signaling has also been shown to be a critical factor for colon CSC malignancy^62,63^. Of note, *Sept4*/ARTS^−/−^ organoids often displayed a striking cystic tumorigenic phenotype, akin to organoids exhibiting hyperactive Wnt signaling, suggesting that ARTS may harbor an additional tumor-suppressing function by indirectly restricting Wnt activity. Thus, it will be of great interest to further explore how the ARTS/XIAP apoptotic module is implicated in driving ISC-dependent tumor initiation, CSC niche maintenance and tumor replenishment.

Finally, we demonstrate that XIAP serves as a target for the pro-apoptotic activity of ARTS in the intestine. One intriguing aspect to consider is that, in this setting, XIAP has been reported to also display non-apoptotic functions as a key mediator of survival pathways^64^. Of note, it has been shown that Paneth cells regulate intestinal NOD2 signaling^64–66^, which plays a vital role in maintaining the intestinal barrier and potentiating protective nuclear factor (NF)-κB activity^13,67^. Indeed, in mice deficient for *XIAP*, certain bacterial infections are unable to be cleared and cells are incapable of activating both the NOD2- and XIAP-mediated NF-κB survival pathways^65,66,68–70^. As such, while we show increased caspase activation in crypts where XIAP is absent or non-functional, it is possible that the increased susceptibility to DSS wounding could be due to a combinatorial effect. Importantly, our findings revealed that when XIAP was compromised, *Sept4/*ARTS^−/−^-dependent phenotypes were reversed. Thus, given that ARTS serves as an apoptotic antagonist of XIAP, one interesting avenue to consider is that ARTS may also function to regulate non-apoptotic XIAP survival pathways.

In summary, we provide important evidence for an apoptotic mechanism that serves to govern the ISC niche and its dependent processes, which offers a platform for the development of targeted therapeutics.

## Methods

### Mice

*Sept4*/ARTS^−/−^, *XIAP*^−/−^, *XIAP*^*ΔRING*^ and *Sept4*^−/−^; *XIAP*^−/−^ mice were generously provided by Prof. Hermann Steller (The Rockefeller University, New York City). All mice, including controls, were on the C57Bl6 background. *Lgr5-EGFP-IRES-creERT2* and *Rosa26-EYFP* mice (Jackson Laboratories) were crossed to *Sept4*/ARTS^−/−^ mice to establish transgenic lineage tracing mice. All mice were bred over multiple generations in the Technion animal facility. Mice were housed under specific pathogen-free conditions at the Technion animal facility with access to food and water ad libitum. All animal studies received institutional ethics approval by the Pre-Clinical Research Authority (PCRA) of the Technion-Israel Institute of Technology and were performed in agreement with this authority’s guidelines (ethics #IL0040115). Mice used in this study were sacrificed using CO_2_ intoxication, in accordance with the PCRA guidelines, at 6–14 weeks of age. For in vivo experiments, mice were sex- and age-matched and randomly assigned to experimental groups. In all DSS experiments, age-matched littermate male mice were used. For induction of Cre-mediated recombination in lineage tracing experiments, mice were administered 100 µl of 1.5 mg/ml Tamoxifen every day for 5 days via intraperitoneal (i.p) injection. In BrdU label retention assays, mice were administered 200 µl of BrdU (GE Healthcare) via i.p injection and harvested 24 h later.

### Intestinal wounding

For IR experiments, mice were administered 14 Gy of abdominal IR, using a moving strip for precise irradiation specifically to the abdomen. In DSS wounding experiments, mice were administered between 2.5–3% (w/v) of DSS as indicated (molecular weight = 40 kDa, Tdb Consultancy AB, Uppsala), which was dissolved in autoclaved drinking water and made available ad libitum. From day 5 onwards the DSS solution was removed and mice were administered fresh drinking water. Weight change and symptoms were assessed every day from the first time point concurrent with DSS administration. At the termination of all wounding experiments, mice were sacrificed ethically according to the PCRA guidelines. Small intestines and colons were extracted, measured and processed for further analyses.

### Immunofluorescence

Intestines extracted from mice were processed for wholemount analysis or embedded in OCT, frozen, sectioned at 5–15 µm and fixed additionally for 10 min in 4% paraformaldehyde (PFA). Human colon samples were provided as paraffin-embedded sections (Oncology Division-Rambam Health Care Campus, Haifa), which were deparaffinized and rehydrated. Antigen retrieval was performed by boiling samples at 95 °C for 20 min. Sections were then blocked and incubated overnight at 4 °C with primary antibodies against: ARTS (Sigma; #SAB3500314 and #A447), β-catenin (Abcam; #ab32572), non-phosphorylated (active) β-Catenin (Cell Signaling; #8814), Ki67 (eBioscience; #14-5698-80), PCNA (Abcam; #ab18197), GFP (Abcam; #ab13970), lysozyme (Dako; #A0099), XIAP (BD; #610762), cleaved caspase-3 (Cell Signaling; #9661), REG4 (R&D Systems; #AF1379), Chromogranin A (Santa Cruz; #sc-376827), villin (Abcam; ab130751) and BrdU (Santa Cruz; #sc-32323). Sections were then incubated with secondary antibodies (Alexa Fluors 488, 546, or 633) for 1 h at room temperature. For TUNEL assays the Click-iT TUNEL Alexa Fluor 594 kit was used according to the manufacturer’s instructions. Sections were then mounted in Vectashield containing 4′,6-diamidino-2-phenylindole (DAPI) and visualized on a Zeiss LSM-880 confocal microscope.

### Intestinal crypt isolation

Following resection, the intestinal lumen was washed extensively and mechanically scraped to remove villi. Intestinal crypt preparations were obtained by incubating tissue in 15 mM EDTA in phosphate-buffered saline (PBS) for 20 min at 4 °C. Crypts were detached from the tissue through vigorous shaking and filtered through a 70 µm filter. For FACS, crypts were further processed in trypsin (5 min at 32 °C) and re-suspended in PBS/10% fetal bovine serum for sorting. Lgr5^+^ cells were sorted by gating for FITC^high^ on forward scatter area. For IF analyses, dissociated crypts were spun-down and re-suspended in 4% PFA for 4 h, before staining. In free-floating crypt assays examining cell death, live crypts were re-suspended in Dulbecco’s modified Eagle medium (DMEM)/F12 medium (Biological Industries) containing l-glutamine (Biological Industries; 1:100), penicillin/streptomycin (Biological Industries; 1:100) and B27 (Rhenium, 1:50). 1 µM STS or dimethyl sulfoxide (DMSO) was applied to freshly dissociated crypts for 2–6 h.

### Intestinal organoid cultures

Organoids were generated from isolated crypts obtained from three pooled intestines. Once extracted, 200–500 crypts were cultured in 50 µl Matrigel and overlaid with DMEM/F12 medium containing l-glutamine (1:100), penicillin/streptomycin (1:100), B27 (1:50), murine EGF (Peprotech; 20 ng/ml), murine Noggin (Peprotech; 100 ng/ml) and human R-Spondin1 (Peprotech; 500 ng/ml), as well as 25 µM Wnt-C59 inhibitor or 1 µM STS, where indicated. Passaging of organoids was performed after 7–9 days of growth by mechanical dissociation with cold PBS. Organoids were split or frozen at a 1:3 ratio. For wholemount staining of organoids ~500 fresh crypts were seeded in 50 µl Matrigel/well in 24-well plates on round coverslips. Following fixation with 2% PFA, incubation with primary and secondary antibodies were performed sequentially overnight at 4 °C. Organoids were then mounted and visualized as described above.

### Western blot and co-IP

For total protein extraction for immunoblotting, crypt-enriched samples were washed with ice-cold PBS, centrifuged (1500 rpm, 5 min at 4 °C), lysed in IP lysis buffer with protease inhibitors and then incubated on ice for 30 min. After centrifugation (14 000 rpm, 15 min at 4 °C), the proteins (supernatant) were removed and quantified (Bradford reagent, BioRad). Equal amounts of proteins were denatured and separated using 12.5% SDS-polyacrylamide gel electrophoresis (SDS-PAGE). Separated proteins were transferred to a nitrocellulose membrane for subsequent immunostaining. Membranes were blocked in 5% non-fat dry milk/PBS-T for 1–2 h, followed by overnight incubation with primary antibody (1:1000). Membranes were then stained with horseradish peroxidase-conjugated secondary antibody and developed using ECL. Staining against tubulin or GAPDH was used a loading control for all western blot experiments. All uncropped blots can be found in Supplementary Fig. 14.

For co-IP, after protein quantification equal amounts of cell lysates were incubated with 5 µg antibody against ARTS (Sigma #SAB3500314) and immunocomplexes were captured on Protein-A/G Agarose beads. Protein samples were resolved on 12.5% SDS-PAGE and electrotransferred to a nitrocellulose membrane. Membranes were blocked in 5% dry skimmed milk in PBS-T and incubated with primary antibodies against ARTS (1:1000) and XIAP (1:1000).

### Real-time PCR

For RT-PCR experiments, three confluent organoid wells or whole intestinal tissues were extracted, weighed and processed immediately in 500 µl or 1 ml TRIzol/100 mg tissue, respectively, for RNA isolation. Whole tissues were homogenized using metal bead-based homogenization (15 Hz, 3 min). Following RNA precipitation, sample concentration was determined for cDNA synthesis (Applied Biosystems). RT-PCR was conducted using the PerfeCTa SYBR Green FastMix (Quanta) and three independent biological and technical triplicates were used. Average values of cycles were normalized relative to the housekeeping GAPDH gene. Forward (f) and reverse (r) primer sequences that were used are listed as follows:

Lgr5 f: 5′-TTGAGGAAGACCTGAAGGC-3′

Lgr5 r: 5′-TCCACTACCGCGATTACC-3′

c-Myc f: 5′-TAGTGCTGCATGAGGAGACA-3′

c-Myc r: 5′-CATCAATTTCTTCCTCATCTTC-3′

Tcf-1 f: 5′-CTGCCTGCTCACAGTTCC-3′

Tcf-1 r: 5′-GGCTCCAGGCCTGTGG-3′

Cyclin D1 f: 5′-ATTGTGCCATCCATGCG-3′

Cyclin D1 r: 5′-TAGATGCACAACTTCTCGGC-3′

Axin2 f: 5′-TGCCGACCTCAAGTGCA-3′

Axin2 r: 5′-ACGCTACTGTCCGTCATGG-3′

Sox9 f: 5′-ACAACGCGGAGCTCAGC-3′

Sox9 r: 5′-GAGTCGGCTTGCAGCG-3′

Wnt3 f: 5′-TGG AAC TGT ACC ACC ATA GAT GAC-3′

Wnt3 r: 5′ ACACCAGCCGAGGCGATG-3′

GAPDH f: 5′-ATGGTGAAGGTCGGTGTGAA-3′

GADPH r: 5′-TCCTGGAAGATGGTGATGGG-3′

### STED microscopy

For super-resolution STED images of ARTS and XIAP, images were acquired on a Leica SP8 scanning confocal microscope (Leica Microsystems, Mannheim, Germany), equipped with a STED module and white-light laser, driven by Leica LASX software. STED super resolution was performed with the 600 nm depletion laser for both fluorophores, using gating with the following settings: gate of 1 ns (Alexa Fluor 488) and 0.3 ns (Alexa Fluor 546). Following acquisition, images were deconvolved using Huygens Professional (Scientific Volume Imaging, The Netherlands), using the CMLE algorithm, with SNR:12 and 40 iterations, as part of the STED workflow.

### Statistical analyses

For all statistical analyses, at least three mice were analyzed of each genotype and the experiments repeated at least twice. Statistical analyses were performed by parametric unpaired two-tailed Student’s *t* test. All quantitations are presented as ± s.e.m, unless otherwise indicated. Images were processed and analyzed using the ImageJ and ZEN programs. Densitometry was performed using Image Studio software.

## Electronic supplementary material

Supplementary Information

## Data Availability

The authors declare that all data supporting the findings of this study are available within this article and its supplementary information files or from the corresponding author upon reasonable request.
