# Supplementary material for: ARTS mediates apoptosis and regeneration of the intestinal stem cell niche
Source: Nat Commun. 2018 Nov 2;9:4582. doi: 10.1038/s41467-018-06941-4 (PMC6214937; doi:10.1038/s41467-018-06941-4)
Supplement: Supplementary file 1 — Supplementary Information [file 41467_2018_6941_MOESM1_ESM.pdf]

Supplementary Information

ARTS Mediates Apoptosis and Regeneration of the Intestinal Stem Cell Niche

Koren et al.

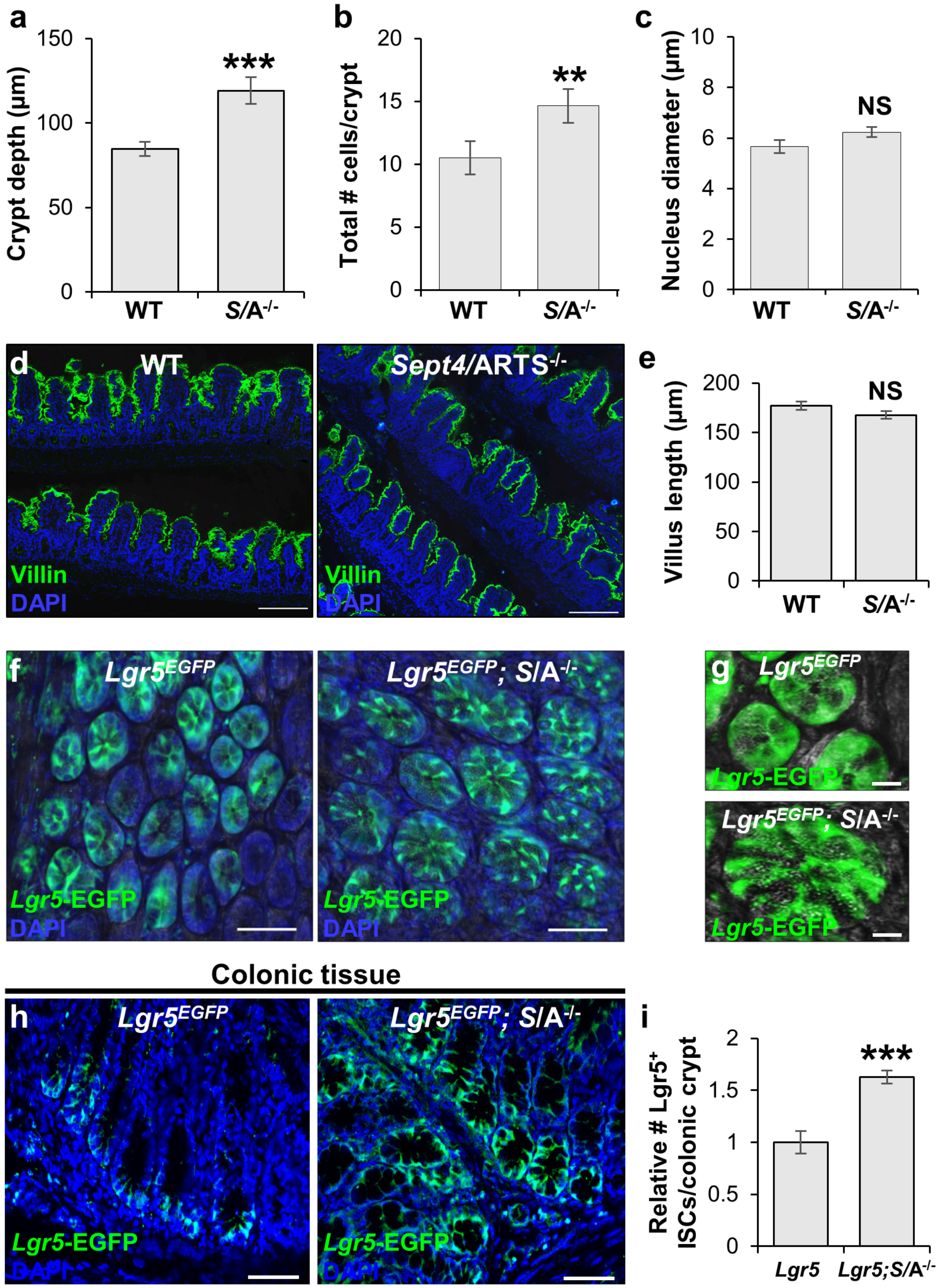

Supplementary Figure 1. Loss of ARTS influences crypt morphology. a-c. Quantifications for (**a**) crypt depth, (**b**) total number of cells per crypt and (**c**) crypt cell nucleus diameter in wild-type (WT) and *Sept4*/ARTS$^{-/-}$ (*S*/A$^{-/-}$) mice. **d.** WT and *S*/A$^{-/-}$ intestinal sections stained against villin. **e.** Quantifications for WT and *S*/A$^{-/-}$ villus length. **f.** Intestinal wholemounts (as shown in Fig. 1a) displaying *Lgr5*-EGFP^{+} intestinal stem cells (ISCs). **g.** Zoom-in on control (*Lgr5^{EGFP}*) and *Lgr5^{EGFP}; S*/A$^{-/-}$ *Lgr5*-EGFP^{+} crypts. Note the high presence of granularity in *Lgr5^{EGFP}; S*/A$^{-/-}$ crypts, indicative of Paneth cells. **h.** *Lgr5*-EGFP^{+} crypts from *Lgr5^{EGFP}* and *Lgr5^{EGFP}; S*/A$^{-/-}$ colonic tissues. **i.** Relative number of Lgr5^{+} ISCs per colonic crypt. All images and quantitations shown are representative of n = 3 mice of each genotype. Error bars represent ± s.e.m. P values were determined using unpaired two-tailed Student's t test where NS = no significance, **$P < 0.01$ and ***$P < 0.005$. All experiments were repeated at least twice. Scale bars: 10µm (g), 50µm (f, h), 100µm (d).

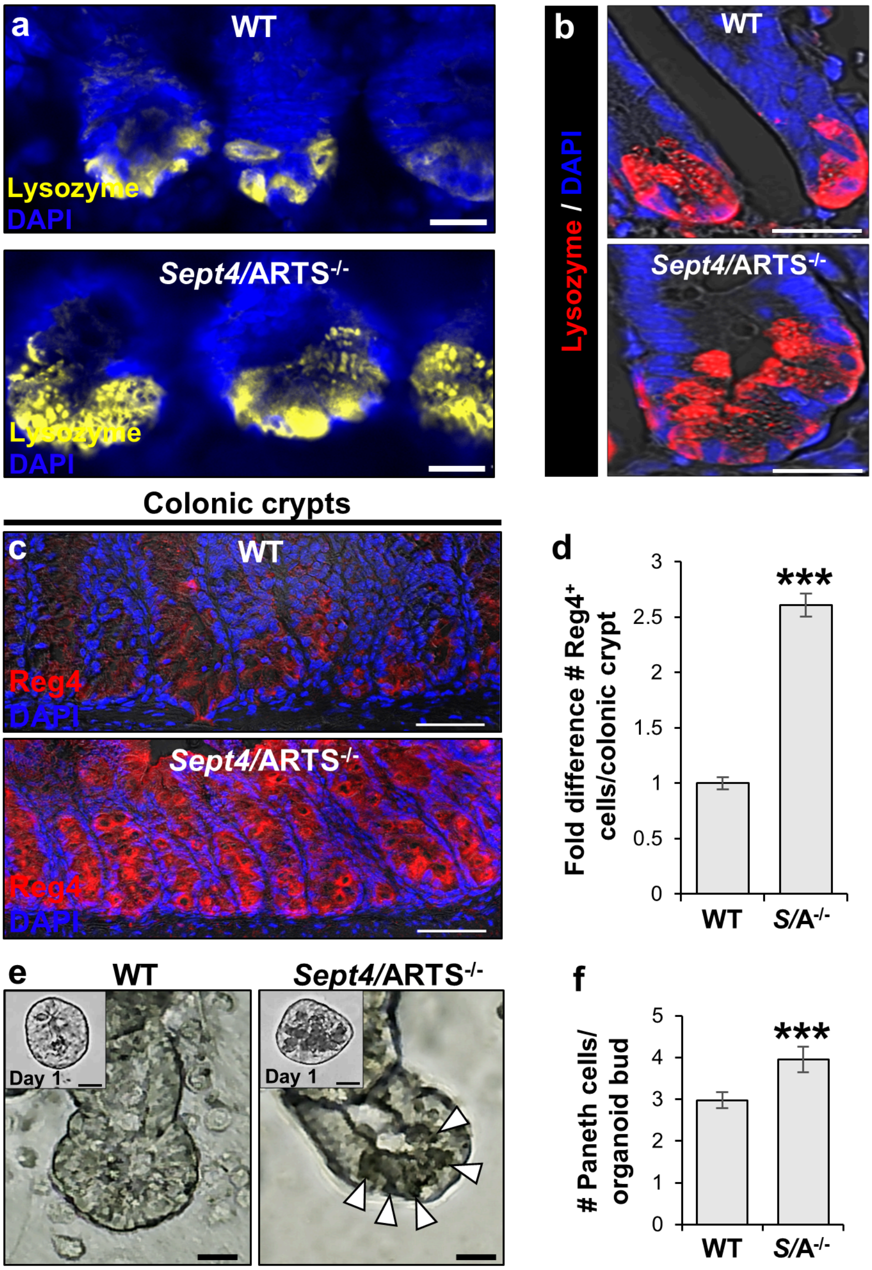

Supplementary Figure 2. ARTS regulates niche cell expansion in the small and large intestinal epithelia. a. Small intestinal crypts stained for lysozyme, showing prominent cytoplasmic lysozyme^{+} granules at the apical surface of Paneth cells. **b.** Small intestinal crypts deleted for *Sept4*/ARTS (*S*/A$^{-/-}$) display greater Paneth cell number and granularity, which can also be visualized by transmitted light. **c.** Colon crypts stained against Reg4, a marker of the epithelial colon niche cell. **d.** Quantification for number of Reg4^{+} niche cells per wild-type (WT) and *S*/A$^{-/-}$ colonic crypt. **e.** *De novo* organoid crypts display Paneth cells in the crypt base that can be observed by brightfield microscopy. Insets show Paneth cells after one day post-seeding isolated crypts. White arowheads indicate Paneth cells, identifiable by granularity and darker color. **f.** Number of Paneth cells per WT and *S*/A$^{-/-}$ organoid crypt *[n* = 3 wells of 3 pooled mice per genotype]. All images and quantitations shown are representative of *n* = 3 mice of each genotype. Error bars represent ± s.e.m. *P* values were determined using unpaired two-tailed Student's *t* test where ***$P < 0.001$. All experiments were repeated at least twice. Scale bars: 10μm (a), 20μm (b, c, e, e inset).

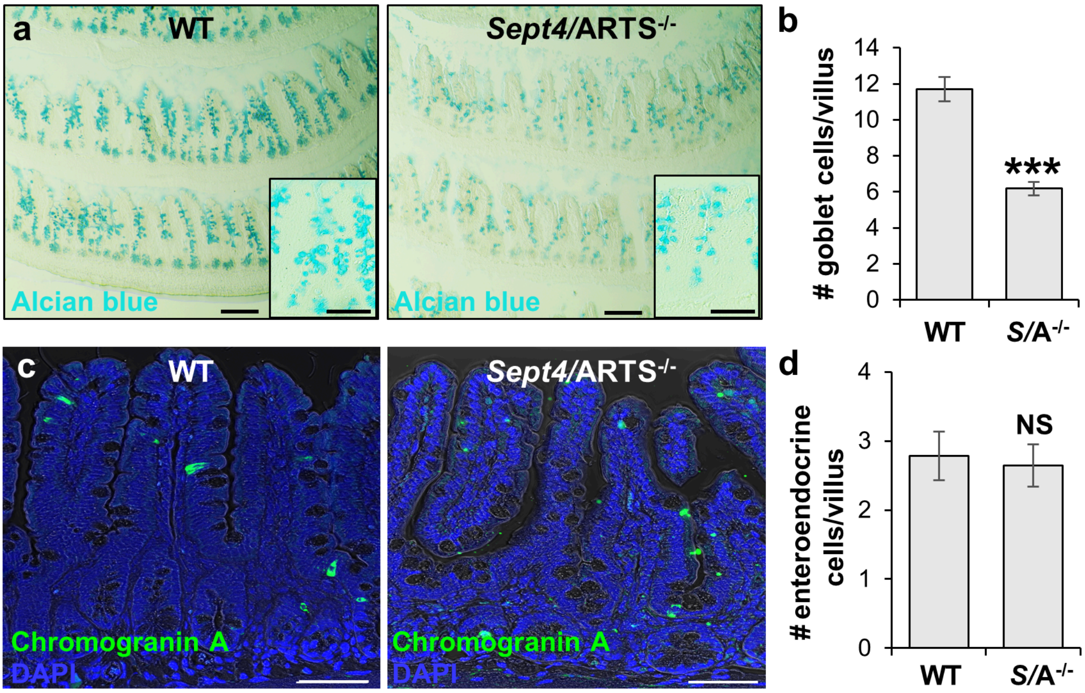

Supplementary Figure 3. Differentiation of the goblet, but not enteroendocrine, cell lineage is affected in *Sept4/*ARTS$^{-/-}$ small intestine. a. Alcian blue staining in wild-type (WT) and *Sept4/*ARTS$^{-/-}$ (*S/*A$^{-/-}$) small intestinal tissues reveals decreased Alcian blue^{+} goblet cells when ARTS is absent. **b.** Quantification of number of goblet (Alcian blue^{+}) cells per villus. **c.** Immunofluorescence showing chromogranin A^{+} enteroendocrine cells in WT and *S/*A$^{-/-}$ villi. **d.** Number of enteroendocrine (chromogranin A^{+}) cells per villus. All images and quantitations shown are representative of n = 4 mice of each genotype. Error bars represent ± s.e.m. P values were determined by unpaired Student's t test where NS = no significance and ***P < 0.001. All experiments were repeated twice. Scale bars: 50μm (a inset, c), 100μm (a).

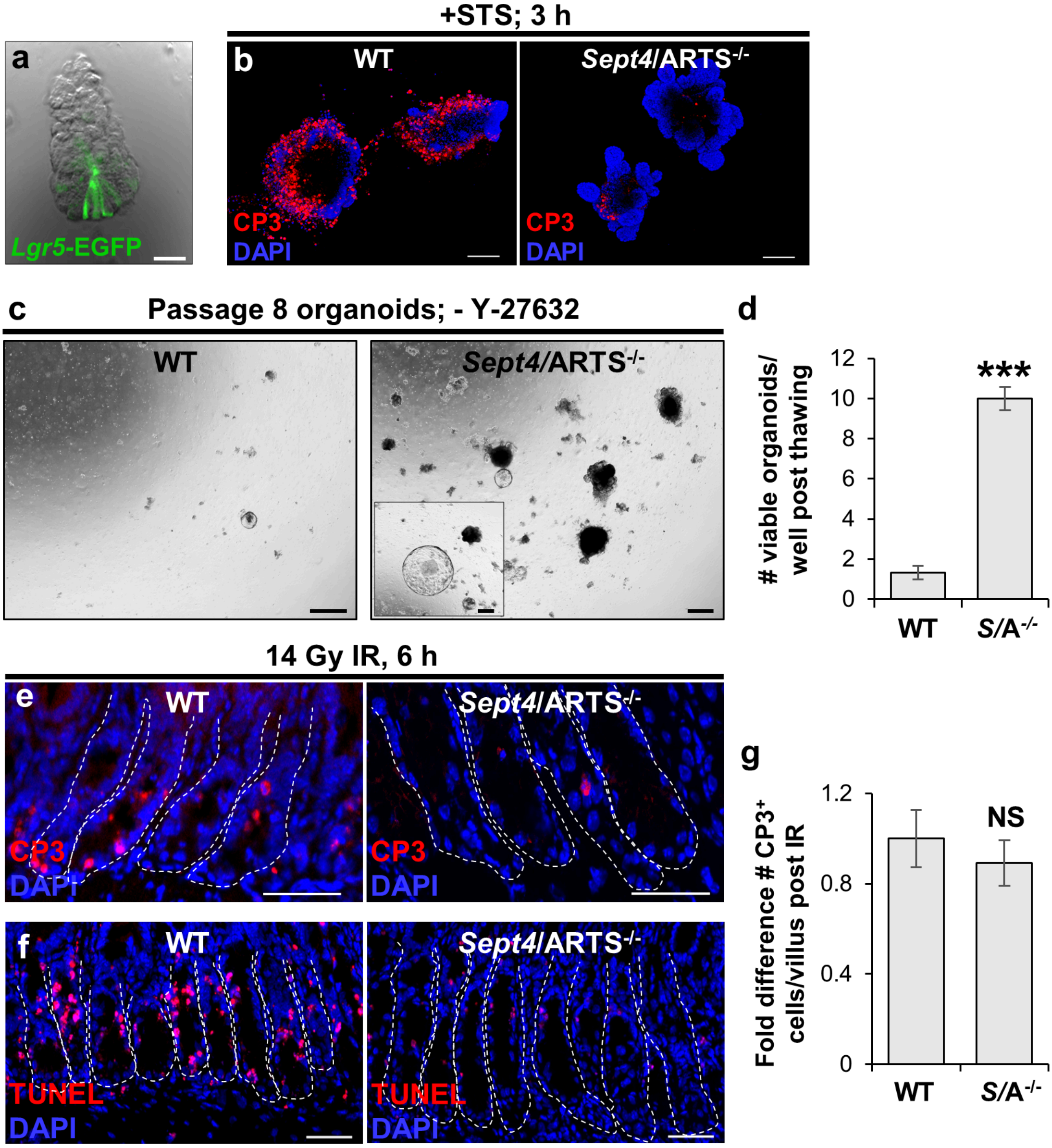

Supplementary Figure 4. Deletion of *Sept4/*ARTS$^{-/-}$ attenuates apoptosis of crypt cells in response to stress and damage. a. Example of isolated and viable *Lgr5*-EGFP^{+} crypt utilized for free-floating crypt apoptotic assays. **b.** Representative images of wild-type (WT) and *Sept4/*ARTS$^{-/-}$ (*S/*A$^{-/-}$) intestinal organoids stained for active caspase-3 (CP3) after treatment with staurosporine (STS) for 3 hours. **c.** *S/*A$^{-/-}$ intestinal organoids thawed from high passage remain viable and expand in the absence of Rock inhibitor (Y-27632) [*n* = 3 wells from 3 pooled mice of each genotype]. Inset shows *S/*A$^{-/-}$ cystic organoid. **d.** Number of viable organoids per well [*n* = 3 wells from 3 mice]. **e-f.** Representative zoomed-out sections of irradiated WT and *S/*A$^{-/-}$ crypts showing (**e**) cleaved CP3^{+} and (**f**) terminal deoxynucleotidyl transferase dUTP nick end labeling (TUNEL)$^{+}$ apoptotic crypt cells. Dashed white lines demarcate intestinal crypts. **g.** Fold difference in number of CP3^{+} cells per WT and *S/*A$^{-/-}$ villus following IR damage [*n* = 4 mice per genotype]. *P* value > 0.05 indicating no significance (NS) and ***$P < 0.0001$ was determined by two-tailed unpaired Student's *t* test. Error bars represent ± s.e.m. All experiments were repeated at least twice. Scale bars: 20μm (a), 50μm (b, e, f), 500μm (c, c inset).

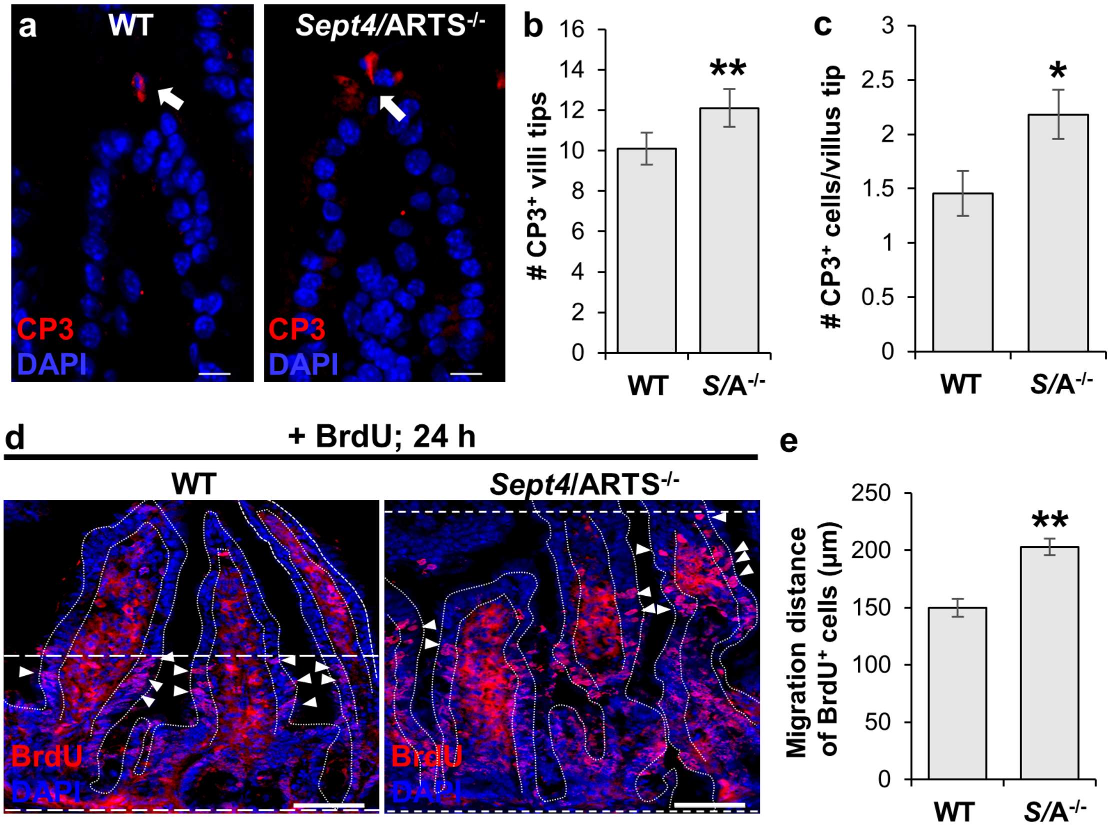

Supplementary Figure 5. Loss of *Sept4*/ARTS accelerates villus tip anoikis and cell migration. a. Wild-type (WT) and *Sept4/*ARTS$^{-/-}$ (*S/*A$^{-/-}$) intestinal tissues demonstrating the presence of cleaved caspase-3 (CP3)$^{+}$ epithelial cells (white arrows) at the villus tip. **b, c.** Quantifications for (**b**) number of CP3^{+} villi tips per section and (**c**) number of CP3^{+} cells per villus tip between WT and *Sept4/*ARTS$^{-/-}$ mice. **d.** 5-Bromo-2'-deoxyuridine (BrdU)$^{+}$ cells in the intestinal epithelia of WT and *Sept4/*ARTS$^{-/-}$ after a 24-hour chase period. White arrowheads indicate BrdU^{+} epithelial cells. White dotted line demarcates epithelial crypt-villi units, while straight white dashed lines mark the base of the crypt to where the furthest BrdU^{+} cell could be detected. Note the presence of BrdU^{+} at the *S/*A$^{-/-}$ villi tips. **e.** Migration distance of BrdU^{+} cells along the crypt-villus axis. All images and quantitations shown are representative of n = 3 mice of each genotype. $^{*}P < 0.05$ and $^{**}P < 0.01$ as determined by unpaired two-tailed Student's t test. All experiments were repeated twice. Error bars represent ± s.e.m. Scale bars, 10μm (a), 100μm (d).

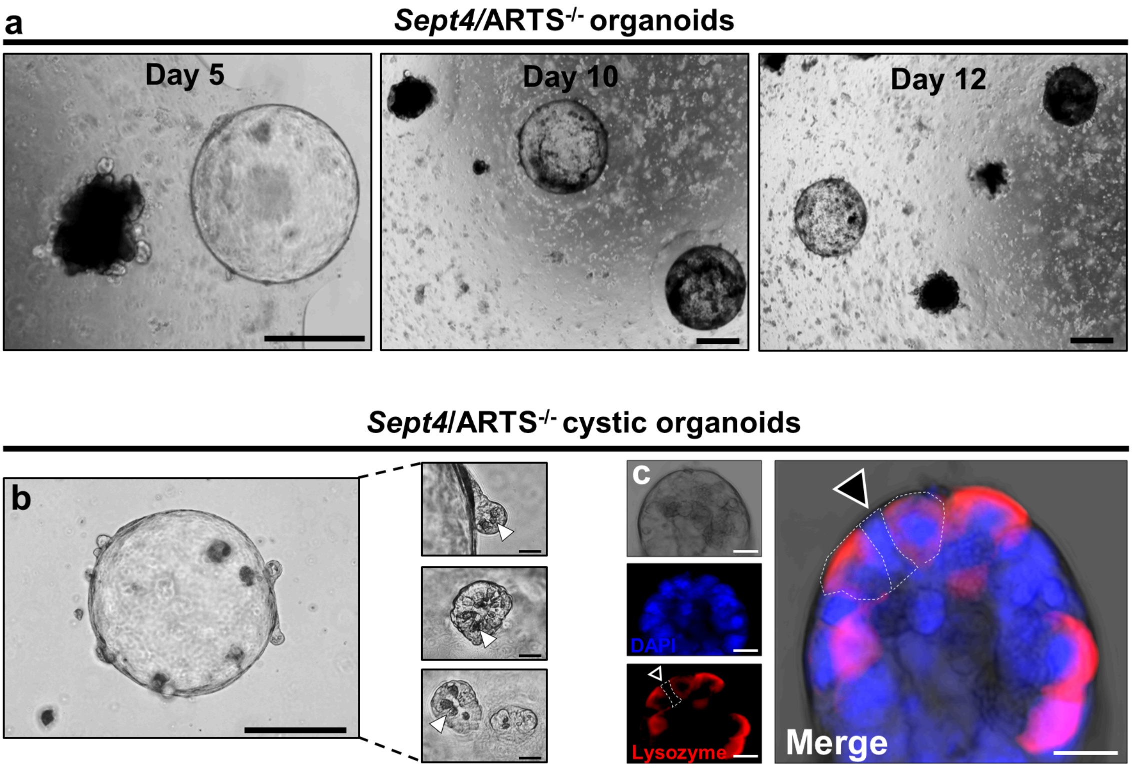

Supplementary Figure 6. Loss of *Sept4*/ARTS leads to formation of cystic organoids. a. *Sept4/*ARTS$^{-/-}$ cystic organoids retain differentiation capacity and become largely differentiated after 10 days in culture. Note the presence of a morphologically normal organoid in close proximity to a cystic organoid (first panel). **b**. Image of cystic *Sept4/*ARTS$^{-/-}$ organoid. Insets display zoomed-in buds that house morphologically normal Paneth cells (white arrowheads) **c.** Cystic organoid bud showing lysozyme^{+} Paneth cells and intercalated lysozyme^{-} crypt base columnar (CBC)-like cell (black arrowhead). Dotted white line demarcates Paneth and CBC cells. Images are representative of n = 3 wells from 3 pooled mice. All experiments were repeated at least twice. Scale bars: 5μm (c), 20μm (b insets), 500μm (a, b).

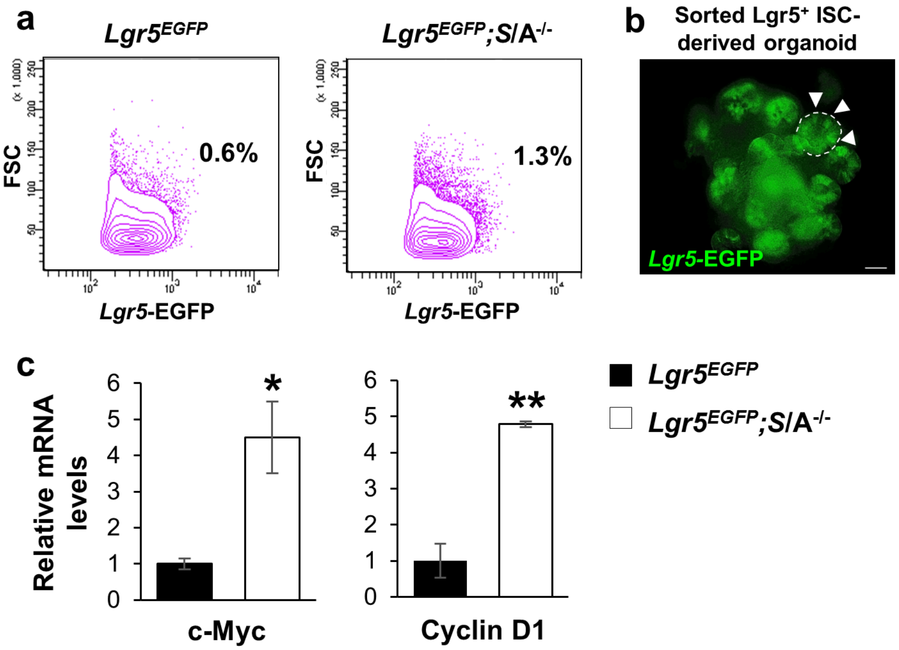

Supplementary Figure 7. Fluorescence-activated cell sorting of Lgr5⁺ stem cells. a. *Lgr5^{EGFP}* reporter mice deleted for *Sept4/*ARTS (*Lgr5^{EGFP}; S/A$^{-/-}$*) mice display greater percentage of sorted *Lgr5*-EGFP^{+} intestinal stem cells (ISCs) than control *Lgr5^{EGFP}* mice [*n* = 3 pooled intestines of each genotype]. **b.** Isolated Lgr5^{+} ISCs can give rise to organoids harboring *Lgr5*-EGFP^{+} crypt cells [*n* = 3 wells from 3 pooled mice per genotype]. Dashed white line demarcates crypt and white arrowheads indicate *Lgr5*-EGFP^{+} ISCs. **c.** Real time-PCR analysis for c-Myc and Cyclin D1 transcripts in sorted Lgr5^{+} ISCs show higher mRNA levels in *Lgr5^{EGFP}; S*/A$^{-/-}$ ISCs [*n* = 3 mice per genotype analyzed in triplicates]. Error bars represent ± s.e.m. **P* < 0.05 and ***P* < 0.01 were determined by two-tailed unpaired Student's *t* test. All experiments were repeated at least twice. Scale bar: 20μm (b).

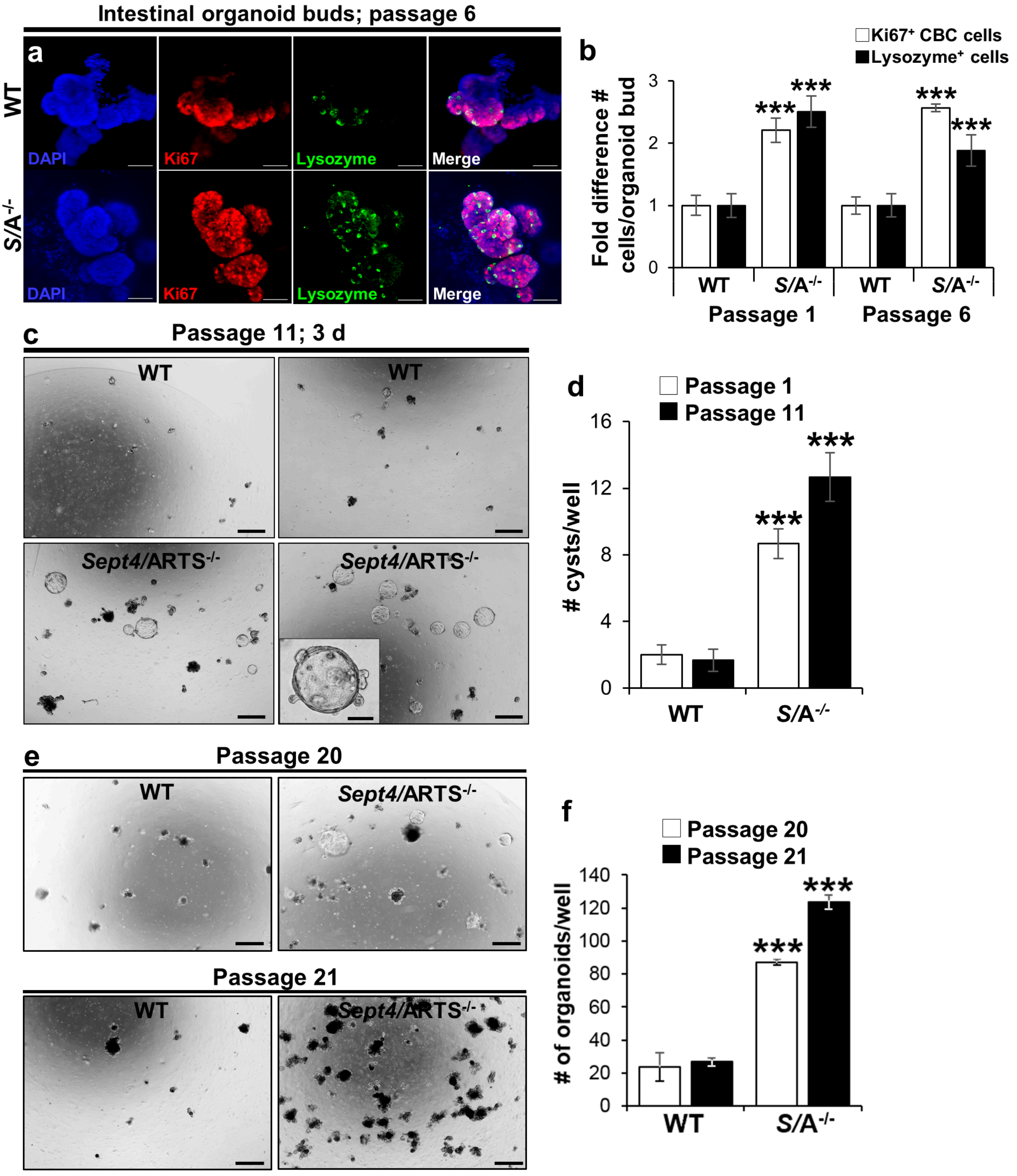

Supplementary Figure 8. Organoids maintain *Sept4*/ARTS$^{-/-}$-dependent phenotypes after multiple passages. a. *Sept4*/ARTS$^{-/-}$ (*S*/A$^{-/-}$) organoids continue to display an augmented intestinal stem cell (ISC) niche after multiple passages. **b.** Number of Ki67^{+} crypt base columnar (CBC) cells and lysozyme^{+} Paneth cells in organoids from passage numbers 1 and 6 [***P < 0.0001 between each genotype for each cell type as determined by two-tailed unpaired Student's t test]. **c.** *S*/A$^{-/-}$ cysts are present at high passage and display budding off the main cystic organoid body. **d.** Number of wild-type (WT) and *S*/A$^{-/-}$ cystic organoids per well. **e.** *S*/A$^{-/-}$ organoids retain high expansion capacity even after 20 passages. **f.** Quantification for number of organoids per well between passage numbers 20 and 21 [***P < 0.0001 comparing between each genotype for each passage number as determined by two-tailed unpaired Student's t test]. All images are representative of n = 3 wells from 3 pooled mice of each genotype. Error bars represent ± s.e.m. All experiments were repeated at least twice. Scale bars: 50µm (a), 200µm (c inset), 500µm (c, e).

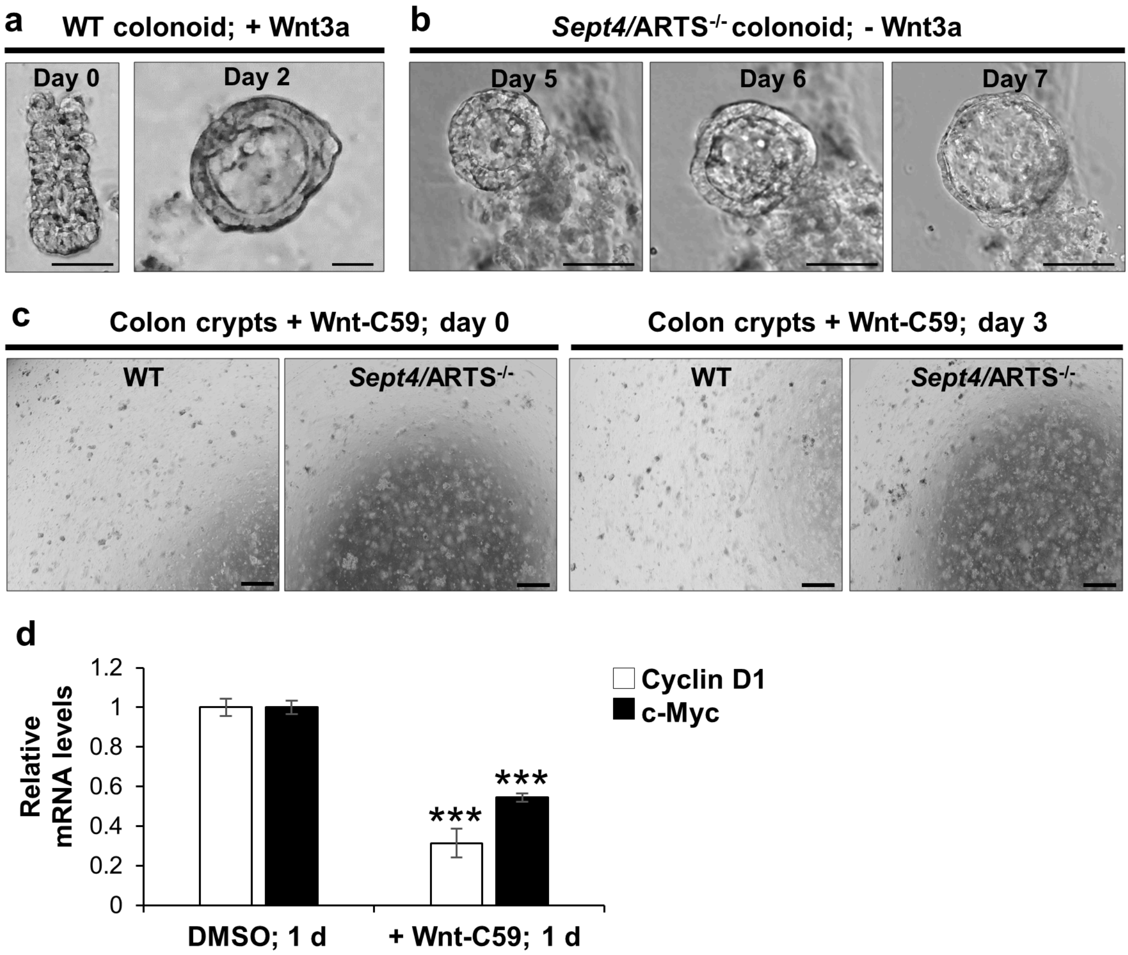

Supplementary Figure 9. Growth of *Sept4/*ARTS$^{-/-}$ colon organoids is Wnt-dependent. a. Isolated colon crypts typically require supplemented exogenous Wnt3a for their growth *ex vivo.* **b.** Development of a colon organoid derived from *Sept4/*ARTS$^{-/-}$ mice in the absence of exogenous Wnt3a. **c.** Wnt-C59-treated wild-type (WT) and *Sept4/*ARTS$^{-/-}$ colon crypts equally depend on Wnt secretion for their growth. **d.** Relative mRNA levels of the Wnt target gene transcripts Cyclin D1 and c-Myc decrease in organoids after 24 hours of Wnt-C59 treatment [n = 5 wells of 3 pooled mice analyzed in triplicates]. Images are representative of n = 3 wells from 3 pooled mice per genotype. Error bars represent ± s.e.m. ***P < 0.005 was determined by two-tailed unpaired Student's t test. All experiments were repeated at least twice. Scale bars: 50μm (a), 200μm (b) and 500μm (c).

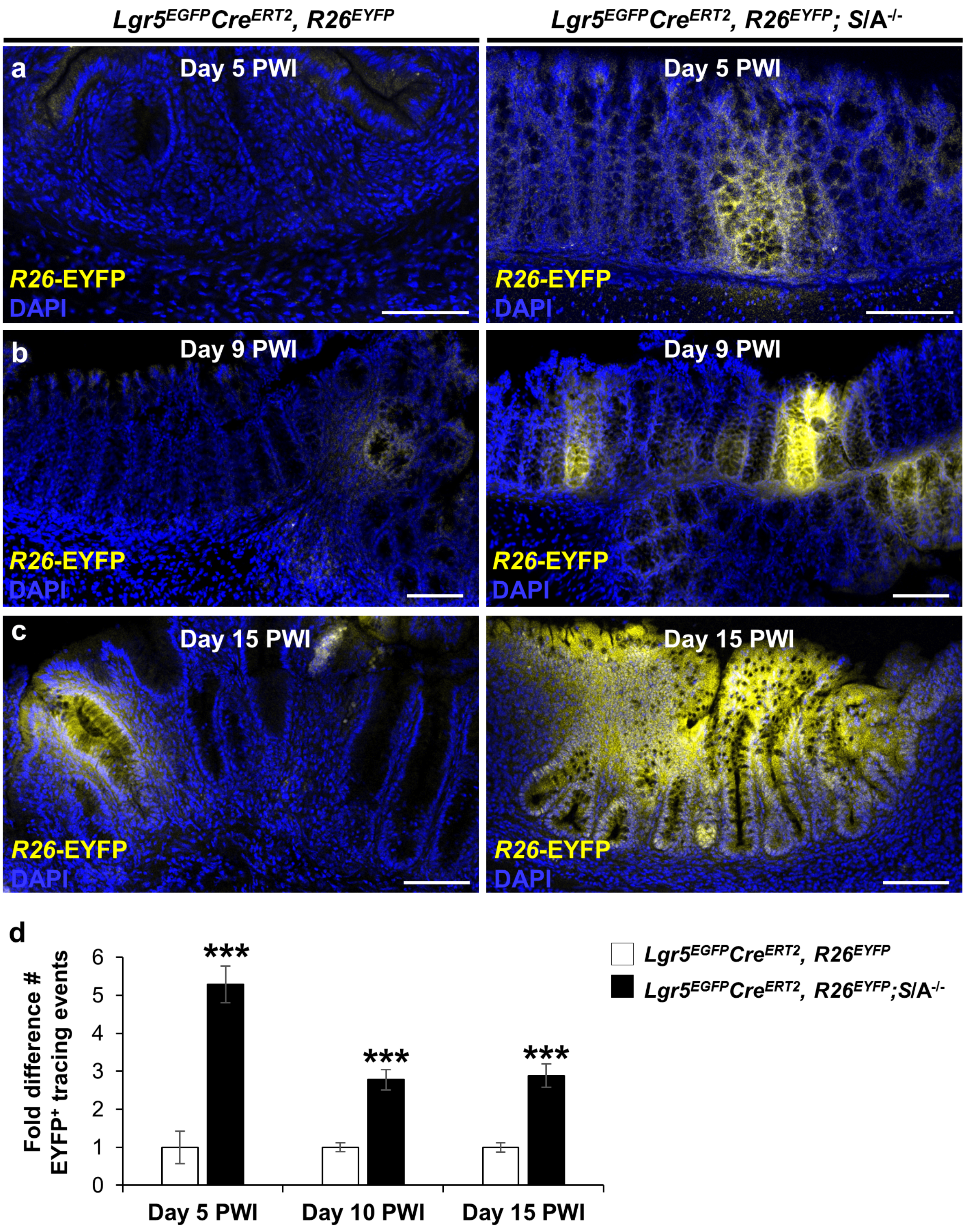

Supplementary Figure 10. Mice deleted for *Sept4*/ARTS display increased regeneration of the colon epithelium post wounding. a-c. Harvested colonic tissues at (**a**) 5 days, (**b**) 9 days and (**c**) 15 days post wound infliction (PWI) by dextran sodium sulfate (DSS), showing stem cell *R26*-EYFP$^+$ progeny forming epithelial ribbons throughout the regenerative response. **d.** Quantifications of fold difference in number of tracing events per time point PWI. All images and quantitations are representative of n = 3 mice per genotype per group. Error bars represent ± s.e.m. ***P < 0.005 as determined by unpaired two-tailed Student's t test. All experiments were repeated twice. Scale bars: 100μm (a-c).

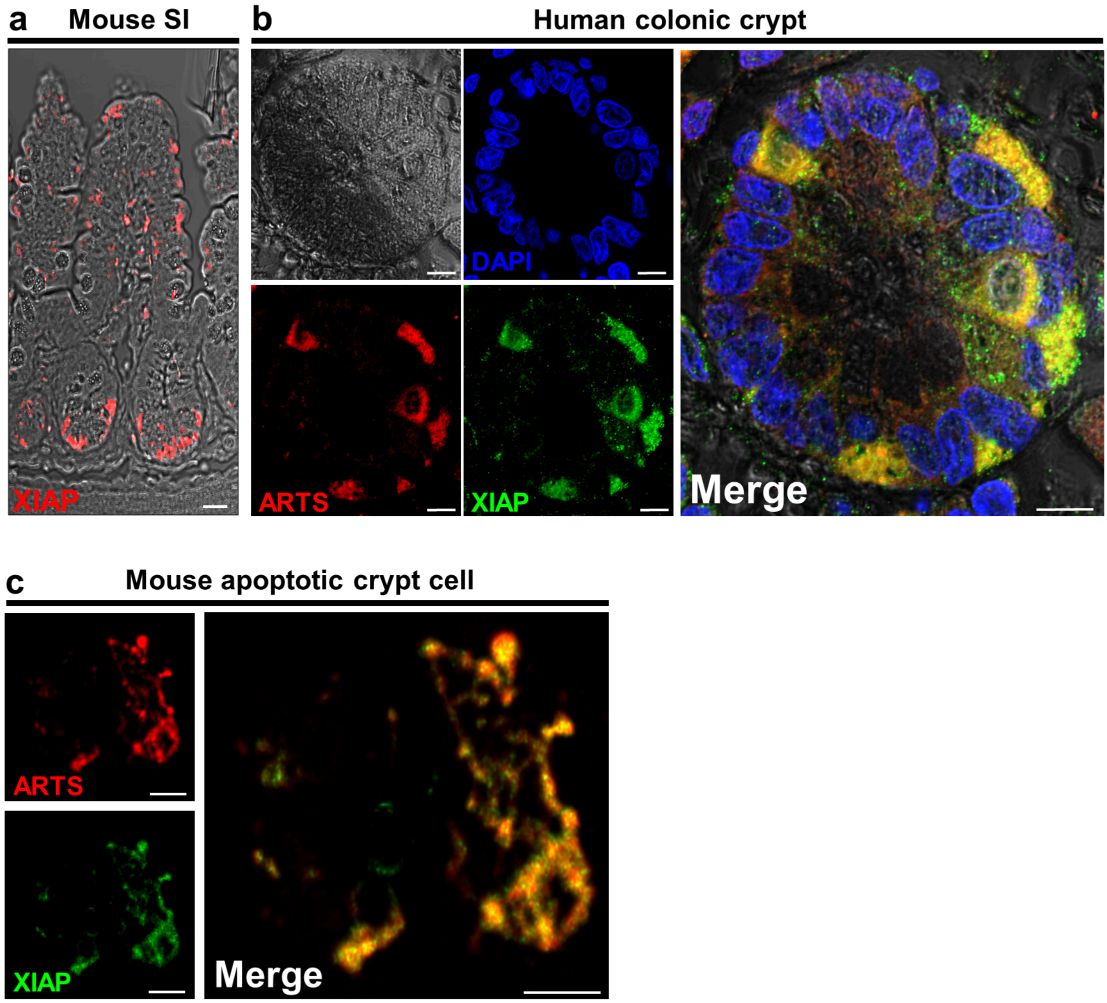

Supplementary Figure 11. ARTS and XIAP co-localize within the intestinal crypt. a. Confocal image of mouse small intestine (SI) stained for XIAP. **b.** Confocal image of human colonic crypt shows crypt cells positive for both ARTS and XIAP. **c.** Super resolution stimulated emission depletion (STED) microscopy image of an apoptotic mouse small intestinal crypt cell shows high degree of co-localization between ARTS and XIAP. All images are representative of *n* = 3 human colons and *n* = 4 mice. All experiments were repeated twice. Scale bars: 3μm (c), 20μm (a, b).

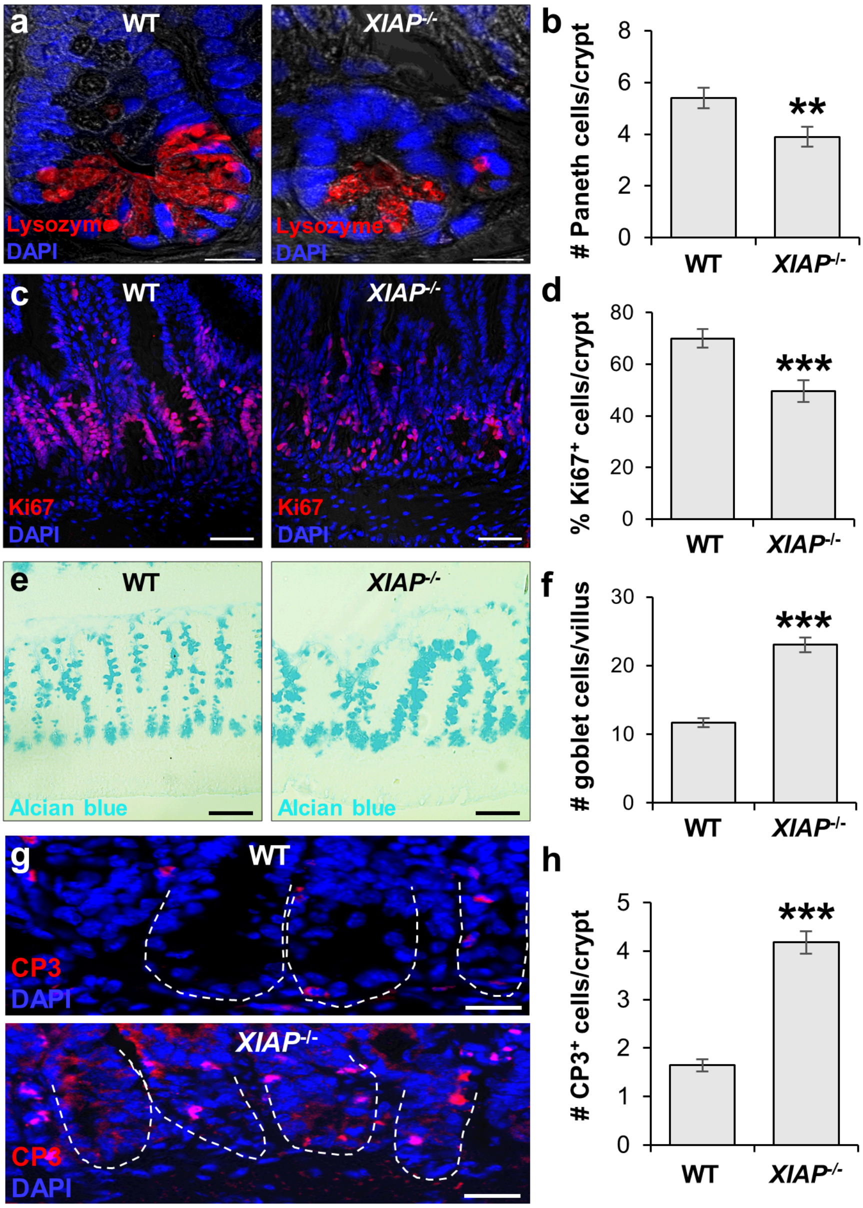

Supplementary Figure 12. Intestinal tissue lacking XIAP displays a reversal of the loss-of-ARTS phenotype. a. Wild-type (WT) and *XIAP*$^{-/-}$ intestinal crypts stained for lysozyme^{+} Paneth cells. **b.** Relative number of lysozyme^{+} Paneth cells per intestinal crypt in WT and *XIAP*$^{-/-}$ mice. **c.** Intestinal crypts stained for Ki67^{+} proliferative cells show less proliferation in *XIAP*$^{-/-}$ intestinal tissue. **d.** Percentage of Ki67^{+} cells per crypt**. e.** Alcian blue staining in WT and *XIAP*$^{-/-}$ small intestinal tissues reveals increased Alcian blue^{+} goblet cells when XIAP is absent. **f.** Number of Alcian blue^{+} goblet cells per villus. **g.** *XIAP*$^{-/-}$ intestinal crypts display increased active caspase-3 (CP3)$^{+}$ cells during homeostasis than WT. White dashed line demarcates crypts. **h.** Number of CP3^{+} cells per WT and *XIAP*$^{-/-}$ crypt. All images and quantitations are representative of n = 3 mice per genotype. P values were determined by two-tailed unpaired Student's t test where $^{**}P < 0.01$ and $^{***}P < 0.001$. Error bars represent ± s.e.m. All experiments were repeated at least twice. Scale bars: 10µm (a), 20µm (g), 50µm (c, e).

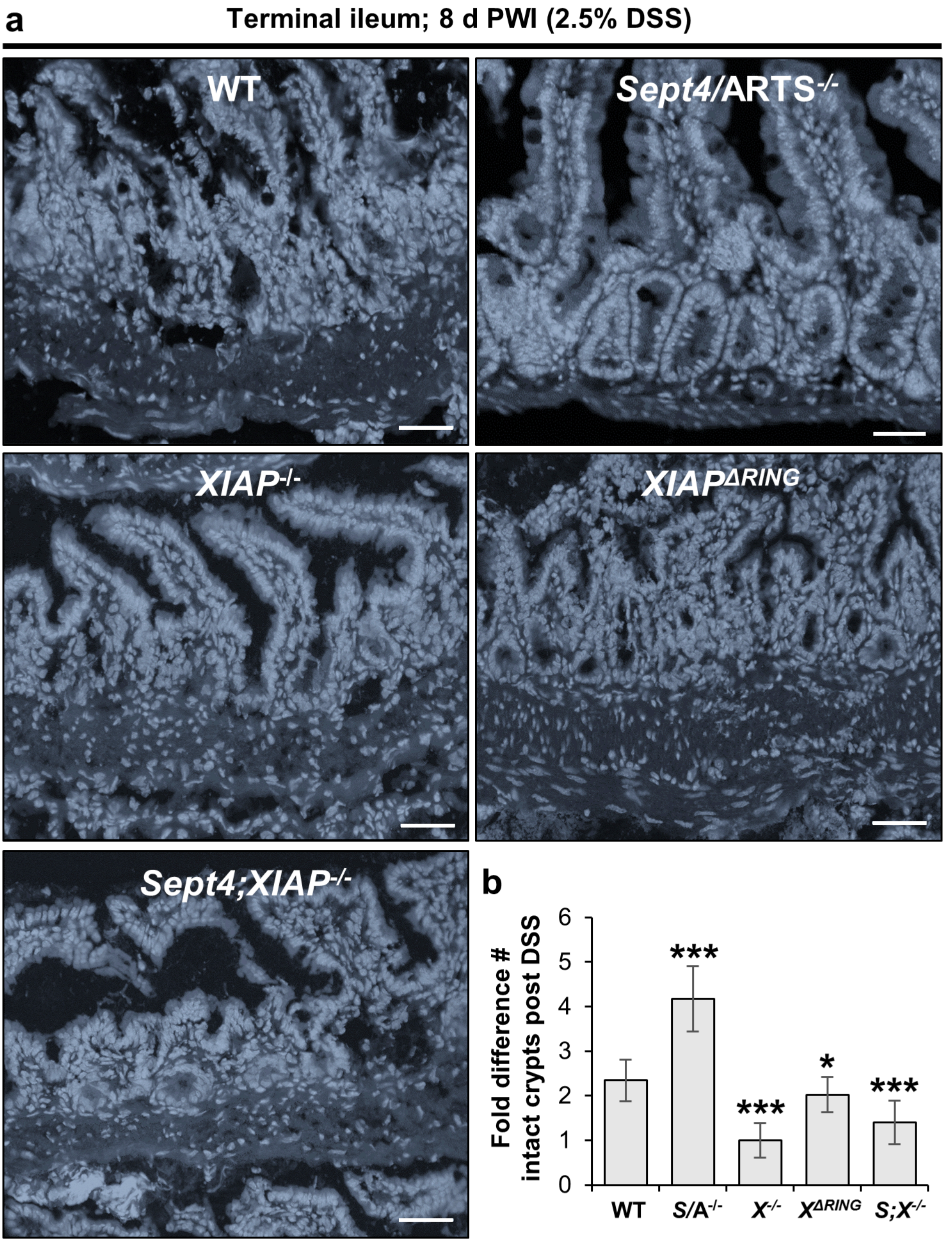

Supplementary Figure 13. Loss of XIAP function exacerbates DSS-mediated injury in the terminal ileum. a. Wild-type (WT), *Sept4/*ARTS$^{-/-}$ (*S*/A$^{-/-}$), *XIAP*$^{-/-}$, *XIAP*$^{\Delta RING}$ and *Sept4*$^{-/-}$*; XIAP*$^{-/-}$ (*S;X*$^{-/-}$) terminal ileum harvested 3 days after a 5-day dextran sodium sulfate (DSS; 2.5% w/v) wounding regime. Nuclei are counterstained with DAPI. *XIAP*$^{-/-}$, *XIAP*$^{\Delta RING}$ and *S;X*$^{-/-}$ tissue display exacerbated inflammation, including distorted tissue architecture, muscle thickening and crypt loss. **b.** Fold difference in number of intact crypts post-DSS treatment. All images and quantifications are representative of n = 3 mice per genotype. P values were determined for each genotype compared to the WT control using unpaired two-tailed Student's t test where *$P < 0.05$ and ***$P < 0.001$. Error bars represent ± s.e.m. All experiments were repeated at least twice. Scale bars: 50μm (a).

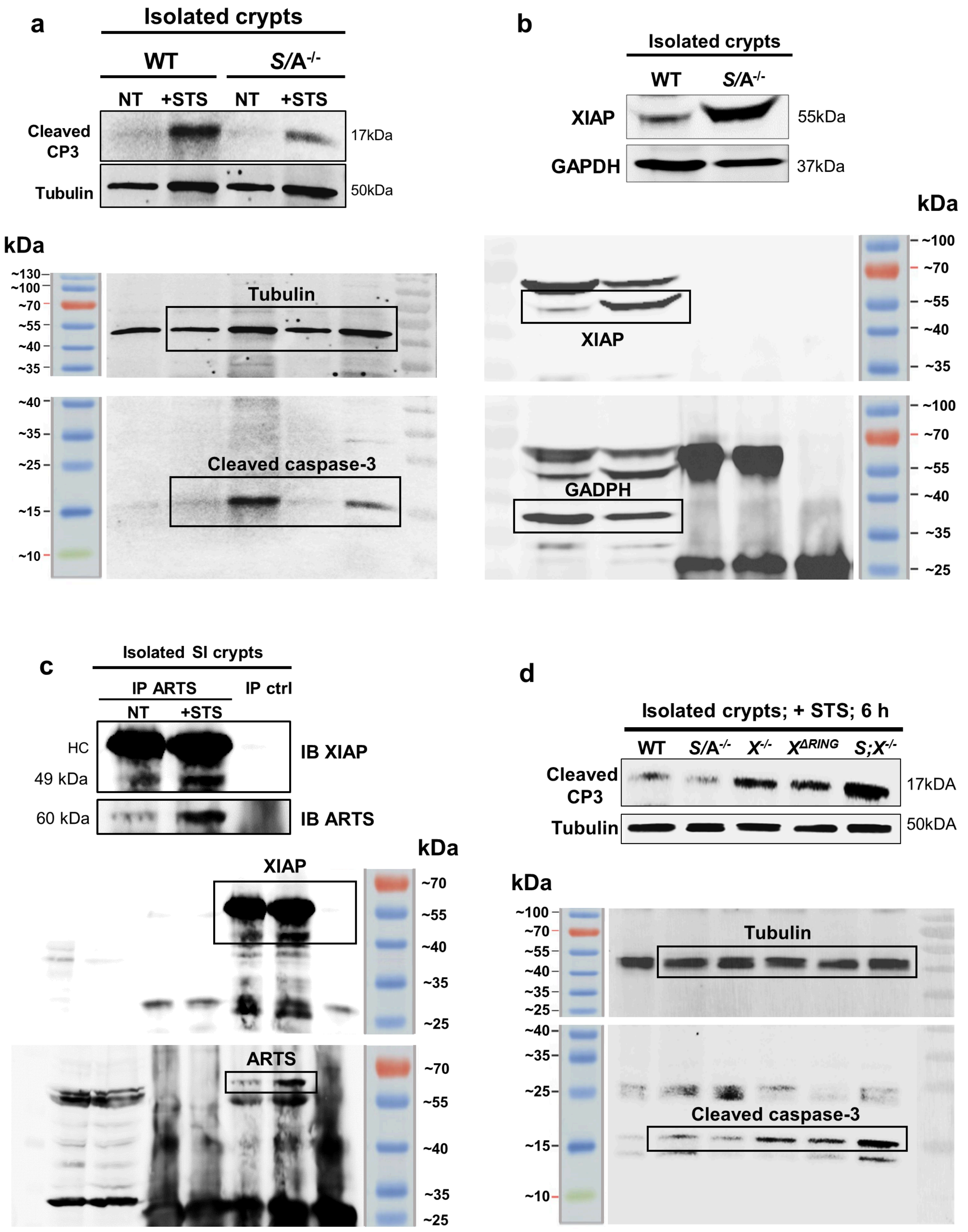

Supplementary Figure 14. Uncropped western blot images. a-d. Uncropped western blot images relating to (**a**) Fig. 3a, (**b**) Fig. 8b, (**c**) Fig. 8e and (**d**) Fig. 8h.
